# Fc-optimized anti-CTLA-4 antibodies increase tumor-associated high endothelial venules and sensitize refractory tumors to PD-1 blockade

**DOI:** 10.1016/j.xcrm.2025.102141

**Published:** 2025-06-03

**Authors:** Lucas Blanchard, Estefania Vina, Jerko Ljubetic, Cécile Meneur, Dorian Tarroux, Maria Baez, Alessandra Marino, Nathalie Ortega, David A. Knorr, Jeffrey V. Ravetch, Jean-Philippe Girard

**Affiliations:** 1 Institut de Pharmacologie et de Biologie Structurale, IPBS, Université de Toulouse, CNRS, UPS, Toulouse, France; 2 Equipe Labellisée LIGUE 2023, Paris, France; 3 Laboratory of Molecular Genetics and Immunology, Rockefeller University, New York, NY, USA; 4 Department of Medicine, Memorial Sloan Kettering Cancer Center, New York, NY, USA

**Keywords:** CTLA-4, ipilimumab, cancer immunotherapy, tumor microenvironment, tumor blood vessels, high endothelial venule, lymphocyte trafficking, tumor-infiltrating lymphocytes, Fc receptors, antibody engineering

## Abstract

The lack of T cells in tumors is a major hurdle to successful immune checkpoint therapy (ICT). Therefore, therapeutic strategies promoting T cell recruitment into tumors are warranted to improve the treatment efficacy. Here, we report that Fc-optimized anti-cytotoxic T lymphocyte antigen 4 (CTLA-4) antibodies are potent remodelers of tumor vasculature that increase tumor-associated high endothelial venules (TA-HEVs), specialized blood vessels supporting lymphocyte entry into tumors. Mechanistically, this effect is dependent on the Fc domain of anti-CTLA-4 antibodies and CD4^+^ T cells and involves interferon gamma (IFNγ). Unexpectedly, we find that the human anti-CTLA-4 antibody ipilimumab fails to increase TA-HEVs in a humanized mouse model. However, increasing its Fc effector function rescues the modulation of TA-HEVs, promotes CD4^+^ and CD8^+^ T cell infiltration into tumors, and sensitizes recalcitrant tumors to programmed cell death protein 1 (PD-1) blockade. Our findings suggest that Fc-optimized anti-CTLA-4 antibodies could be used to reprogram tumor vasculature in poorly immunogenic cold tumors and improve the efficacy of ICT.

## Highlights

• Anti-CTLA-4 antibodies increase TA-HEVs through Fc effector function
• CD4^+^ T cells are important for increase of TA-HEVs during anti-CTLA-4 therapy
• Ipilimumab requires Fc-engineering to increase TA-HEVs in humanized mice
• Fc-optimized anti-CTLA-4 antibodies sensitize cold tumors to PD-1 blockade

## Graphical abstract

**Figure:**
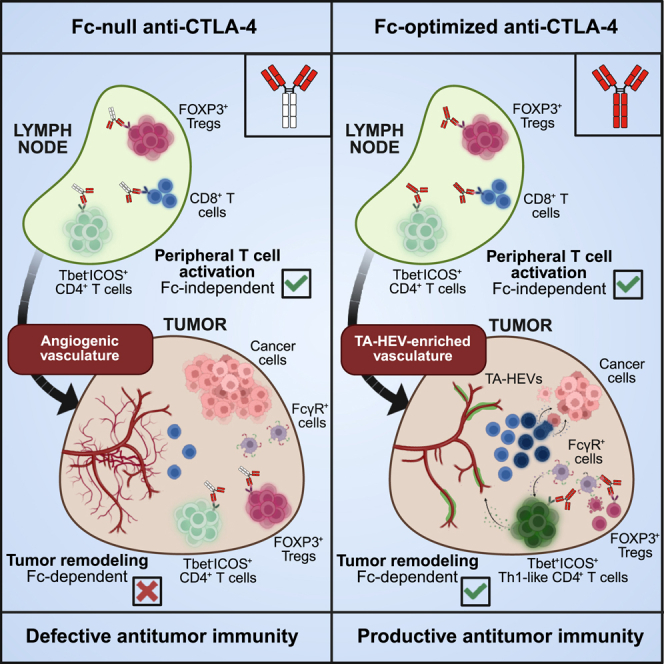

## Teaser

Blanchard et al. reveal that Fc-optimized anti-CTLA-4 antibodies remodel tumor vasculature and increase tumor-associated high endothelial venules (TA-HEVs) that support T cell recruitment into tumors. Using a humanized mouse model, they show that an Fc-optimized version of ipilimumab increases TA-HEVs and sensitizes refractory tumors to PD-1 blockade.

## Introduction

Immune checkpoint therapy (ICT) targeting cytotoxic T lymphocyte antigen 4 (CTLA-4) and programmed cell death protein 1 (PD-1) has provided remarkable clinical responses over the past decade, but these benefits are restricted to a subset of patients with cancer.^1^^,^^2^ The lack of T cell infiltration into tumors is a major limitation to successful cancer immunotherapy,^3^^,^^4^ and inversely, the presence of T cells into tumors correlates with improved clinical responses,^5^^,^^6^^,^^7^ suggesting that therapeutic strategies increasing the abundance of T cells into tumors could improve the efficacy of the treatment.

In lymph nodes and other secondary lymphoid organs, lymphocyte trafficking is mediated by specialized blood vessels called high endothelial venules (HEVs).^8^ HEVs express sulfated sialomucins that are recognized by the lymphocyte homing receptor L-selectin (CD62L) and the HEV-specific antibody MECA-79.^8^ MECA-79^+^ HEV-like blood vessels (tumor-associated HEVs, TA-HEVs) are frequently observed in areas of intense lymphocyte infiltration in human and mouse tumors.^9^^,^^10^ Recent studies revealed that TA-HEVs are major sites of lymphocyte extravasation into tumors.^11^ TA-HEVs derive from tumor post-capillary venules and exhibit a bifunctional phenotype characterized by the co-expression of MECA-79^+^ HEV sialomucins and the inflammatory P-selectin (CD62P).^11^^,^^12^ Furthermore, TA-HEVs are associated with the formation of intratumoral immune hubs and tertiary lymphoid structures that are known to be enriched in patients responding to ICT.^12^^,^^13^^,^^14^ Therefore, there is a growing interest in modulating TA-HEVs in patients to promote the recruitment of lymphocyte into tumors and improve the efficacy of cancer immunotherapy.^10^

Under physiological conditions, dendritic cells maintain the mature HEV phenotype in lymph nodes by stimulating the lymphotoxin-β receptor (LTβR).^15^ In line with this, our group and others have shown that stimulation of LTβR with its ligands lymphotoxin-α_1_β_2_ and LIGHT (TNFSF14),^14^^,^^16^^,^^17^ or with agonistic antibodies,^11^^,^^12^^,^^18^ increases the formation of TA-HEVs, promotes T cell infiltration, and synergizes with ICT. However, the clinical translation of this therapeutic strategy is compromised by the wide expression of the LTβR in both normal and malignant tissues, as well as the high risk of side effects because of the central role of this receptor in lymphoid tissue neogenesis during chronic inflammatory and autoimmune diseases.^19^ Unexpectedly, we recently found that ICT with the anti-mouse CTLA-4 monoclonal antibody (mAb) 9D9 increases the proportion of TA-HEVs in mouse preclinical tumor models.^11^ In contrast, we did not observe changes in TA-HEVs after treatment with the anti-mouse PD-1 mAb RPM1-14.^11^ These results suggested that anti-CTLA-4 antibodies may have a specific capacity to increase TA-HEVs. However, the molecular mechanisms behind this unexpected activity of anti-CTLA-4 antibodies, and whether currently available human anti-CTLA-4 antibodies could modulate TA-HEVs, remain to be defined.

CTLA-4 negatively regulates T cell activation by competing with the costimulatory receptor CD28 for binding to B7 ligands (B7.1/CD80 and B7.2/CD86) on antigen-presenting cells.^20^ Blockade of CTLA-4 with antibodies thus directly activates T cells, resulting in the expansion of effector T cells and the broadening of the T cell receptor repertoire.^21^^,^^22^ CTLA-4 also limits CD4^+^ T cell differentiation potential,^23^ and anti-CTLA-4 antibodies have the capacity to induce non-canonical effector CD4^+^ T cell subsets, including Tbet^+^ICOS^+^ Th1-like CD4^+^ T cells,^22^^,^^24^^,^^25^ which are important for treatment efficacy.^26^ Additionally, several preclinical studies in mouse models demonstrated that Fc-dependent depletion of tumor-infiltrating FOXP3^+^ regulatory T cells (Tregs), which highly express CTLA-4, co-defines the efficacy of anti-CTLA-4 therapy.^27^^,^^28^^,^^29^^,^^30^^,^^31^^,^^32^ However, there is currently no definitive evidence that approved human anti-CTLA-4 antibodies (ipilimumab and tremelimumab) deplete intratumoral Tregs in patients with cancer,^33^^,^^34^ which is likely to be due to the isotype format of these antibodies that were initially selected for their ability to block CTLA-4 and not to deplete Tregs.^22^^,^^35^ More recently, the Fc-dependent activation/reprogramming of myeloid cells has also been proposed to be important for the efficacy of anti-CTLA-4 therapy.^36^^,^^37^^,^^38^ Overall, the mechanisms of action of anti-CTLA-4 antibodies are still incompletely understood, and discrepancies can exist between observations made in conventional mouse models and patients. A better understanding of such mechanisms is required for the development of next-generation anti-CTLA-4 antibodies with increased efficacy and to guide their rationale combination with other therapies.

Here, we investigate the mechanisms by which anti-CTLA-4 antibodies modulate TA-HEVs and T cell-dependent antitumor immunity in preclinical mouse tumor models. We demonstrate that the antibody Fc effector function and CD4^+^ T cells are both required for the remodeling of tumor blood vessels and the increase of TA-HEVs during anti-CTLA-4 therapy. Using a humanized mouse model, we additionally show that an Fc-optimized version of the human anti-CTLA-4 antibody ipilimumab successfully increases TA-HEVs and sensitizes recalcitrant tumors to PD-1 blockade, validating the translational relevance of our findings.

## Results

### Anti-CTLA-4 antibodies increase the proportion of TA-HEVs through Fc-dependent mechanisms

**Figure 1:**
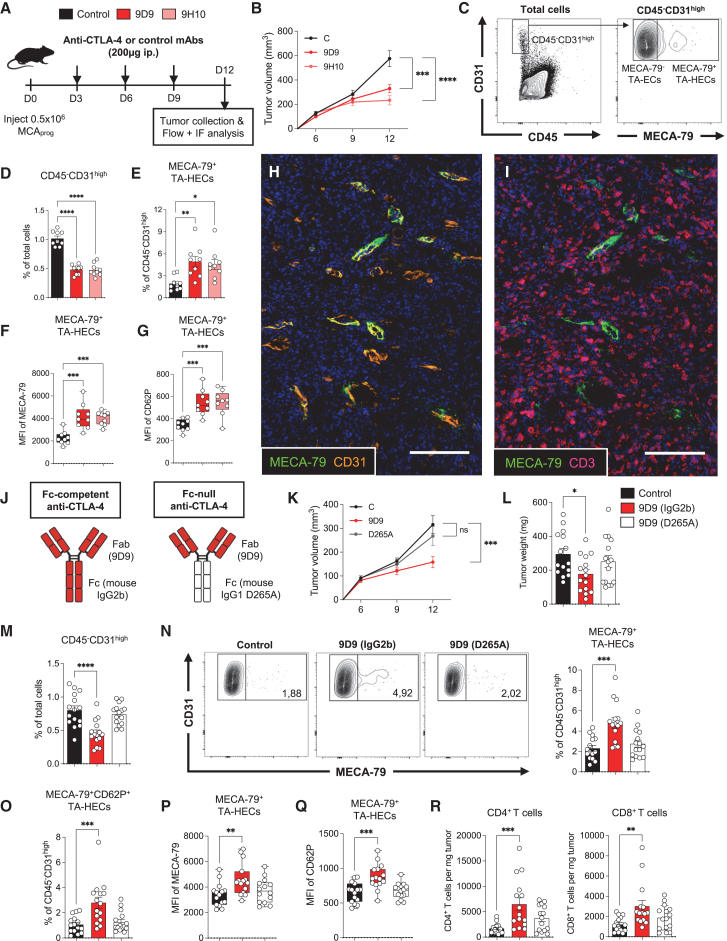
Anti-CTLA-4 antibodies remodel tumor vasculature and increase TA-HEVs through Fc-dependent mechanisms (A) Treatment schedule. i.p., intraperitoneal; IF, immunofluorescence; control, isotype control antibodies. (B) Mean tumor growth (*n* = 9 mice per group; data pooled from two independent experiments). (C) Gating strategy for CD45^−^CD31^high^ endothelial cells and MECA-79^+^ TA-HECs. (D and E) Frequencies of CD45^−^CD31^high^ endothelial cells and TA-HECs. Each symbol represents an individual mouse (*n* = 9, two independent experiments). (F and G) Mean fluorescence intensity (MFI) of MECA-79 and CD62P in TA-HECs, quantified by flow cytometry. Each symbol represents an individual mouse (*n* = 9, two independent experiments). (H and I) Immunofluorescence of MCA_prog_ tumor (day 12) following treatment with 9D9 mouse anti-CTLA-4 antibody. Selected markers are presented. Scale bars: 100 μm. (J) Schematics of the two distinct 9D9 anti-CTLA-4 antibodies used in the study. (K and L) Mean tumor growth and tumor weights in individual mice (*n* = 15 mice per group; data pooled from three independent experiments). (M–O) Frequencies of CD45^−^CD31^high^ endothelial cells, TA-HECs, and MECA-79^+^CD62P^+^ TA-HECs. Each symbol represents an individual mouse (*n* = 15, three independent experiments). Representative dot plots of TA-HECs are shown (N). (P and Q) MFI of MECA-79 and CD62P in TA-HECs, quantified by flow cytometry. Each symbol represents an individual mouse (*n* = 15, three independent experiments). (R) Numbers of tumor-infiltrating CD4^+^ and CD8^+^ T cells. Each symbol represents an individual mouse (*n* = 15, three independent experiments). Data are shown as mean ± SEM. All *p* values were determined by one-way ANOVA with Tukey’s multiple comparison test, except for (B) and (K) for which *p* values were determined by two-way ANOVA with Dunnett’s multiple comparison test. See also Figures S1 and S2.

We first compared side by side the vascular-modulating properties of anti-CTLA-4 mAbs 9D9 and 9H10, using a methylcholanthrene (MCA)-induced mouse tumor model (MCA_prog_) in which TA-HEVs spontaneously develop and mediate lymphocyte infiltration into tumors.^11^ Tumors were collected at a time point of increased tumor control (Figures 1A, 1B, S1A, and S1B) for flow cytometry (Figure 1C)^39^ and immunofluorescence analyses. We found that treatment with either 9D9 or 9H10 induced a 2-fold reduction in the frequency and number of total CD45^−^CD31^high^ tumor endothelial cells (Figures 1D and S1C), indicative of a potent anti-angiogenic activity. The number of MECA-79^−^ tumor-associated endothelial cells (TA-ECs) was specifically reduced, while the number of MECA-79^+^ TA-HEV endothelial cells (TA-HECs) was unaffected (Figure S1D), resulting in a significant increase of their frequency (Figure 1E). In addition, the mean fluorescence intensity (MFI) of MECA-79 and CD62P was significantly increased on MECA-79^+^ TA-HECs (Figures 1F and 1G), as was the frequency of TA-HECs co-expressing MECA-79 and CD62P (Figure S1E). Immunofluorescence staining revealed that MECA-79^+^CD31^+^ TA-HEVs were surrounded by numerous CD3^+^ T cells after treatment (Figures 1H, 1I, S1F, and S1G). Finally, we found that intratumoral injection of a 4-fold reduced dose of 9H10 was sufficient to increase MECA-79^+^ TA-HECs and inhibit tumor growth (Figures S1H–S1J). Collectively, these results indicate that anti-CTLA-4 antibodies reinforce the phenotype of TA-HECs and increase their abundance in the tumor microvasculature by selectively eliminating non-HEV MECA-79^−^ TA-ECs.

Previous studies demonstrated that Fc-dependent mechanisms are involved in the antitumor activity of mouse anti-CTLA-4 antibodies,^27^^,^^28^^,^^29^^,^^30^^,^^31^^,^^32^^,^^37^ including 9D9 (mouse IgG2b) and 9H10 (Syrian hamster IgG2b) mAbs. Effector responses induced by IgG antibodies are dependent on their binding affinities for the activating and inhibitory Fcγ receptors (FcγRs),^40^^,^^41^ and the ratio of activating-to-inhibitory receptor binding (A/I) determines the relative Fc effector function of IgG isotypes (Figure S2A). We evaluated the effects on tumor vasculature of an Fc-null 9D9 that does not bind FcγRs (mouse IgG1 D265A Fc variant) in comparison with parental 9D9 (Figure 1J). Remarkably, absence of Fc effector function prevented the effects of 9D9 on tumor control (Figures 1K, 1L, S2B, and S2C) and on CD45^−^CD31^high^ tumor endothelial cells, MECA-79^−^ TA-ECs, and MECA-79^+^ TA-HECs (Figures 1M–1Q and S2D). We obtained similar results when the original Fc of 9H10 was switched to mouse IgG1 Fc (Figures S2E–S2H), which preferentially binds the inhibitory FcγRIIb (Figure S2A). Finally, we found that lack of TA-HEV modulation during treatment was associated to defects in the accumulation of CD4^+^ and CD8^+^ T cells into tumors (Figure 1R). Altogether, our results indicate that anti-CTLA-4 antibodies remodel tumor vasculature and increase TA-HEVs through Fc-dependent mechanisms.

### Tbet^+^ICOS^+^ Th1-like CD4^+^ T cells correlate with TA-HEVs during anti-CTLA-4 therapy

**Figure 2:**
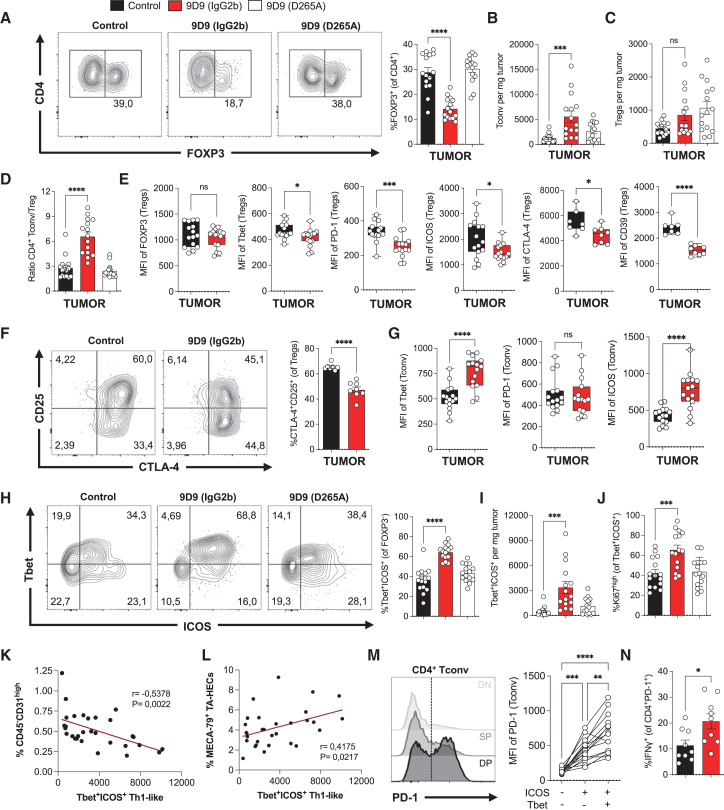
Tbet^+^ICOS^+^ Th1-like CD4^+^ T cells correlate with TA-HEVs during anti-CTLA-4 therapy (A–C) Frequency of FOXP3^+^ Tregs and numbers of FOXP3^−^ Tconv and FOXP3^+^ Tregs in tumors following indicated treatments. Representative dot plots of FOXP3^+^ Tregs are shown (A). Each symbol represents an individual mouse (*n* = 15, three independent experiments). (D) Ratio of Tconv to Treg cell numbers in tumors following indicated treatments. Each symbol represents an individual mouse (*n* = 15, three independent experiments). (E) Mean fluorescence intensity (MFI) of selected markers in Tregs, quantified by flow cytometry. Each symbol represents an individual mouse (FOXP3, Tbet, PD-1, and ICOS, *n* = 15, three independent experiments; CTLA-4 and CD39, *n* = 7–8, two independent experiments). (F) Frequency of CTLA-4^+^CD25^+^ Tregs in tumors following indicated treatments. Representative dot plots are shown. Each symbol represents an individual mouse (*n* = 7–8, two independent experiments). (G) MFI of selected markers in Tconv, quantified by flow cytometry. Each symbol represents an individual mouse (*n* = 15, three independent experiments). (H and I) Frequency and number of Tbet^+^ICOS^+^ Th1-like CD4^+^ T cells in tumors following indicated treatments. Representative dot plots are shown (H). Each symbol represents an individual mouse (*n* = 15, three independent experiments). (J) Frequency of Ki67^high^ cells in Tbet^+^ICOS^+^ Th1-like CD4^+^ T cells in tumors following indicated treatments. Each symbol represents an individual mouse (*n* = 15, three independent experiments). (K and L) Correlation analysis between the number of tumor-infiltrating Tbet^+^ICOS^+^ Th1-like CD4^+^ T cells and the frequencies of CD45^−^CD31^high^ endothelial cells or MECA-79^+^ TA-HECs. Each symbol represents an individual mouse, and simple linear regression lines are shown in red (data pooled from six independent experiments; *n* = 30 mice treated with 9D9 or 9H10 anti-CTLA-4 antibodies). *p* values and Pearson’s r were determined by Pearson correlation test. (M) Histograms showing expression of PD-1 in Tbet^−^ICOS^−^ (DN), Tbet^−^ICOS^+^ (SP), and Tbet^+^ICOS^+^ (DP) FOXP3^−^ Tconv from MCA_prog_ tumors (day 12) following treatment with 9D9 anti-CTLA-4 antibody, quantified by flow cytometry. MFI of PD-1 is quantified in indicated subsets. Each symbol represents an individual mouse (*n* = 15, three independent experiments). (N) Frequency of IFNγ^+^ cells in CD44^+^PD-1^+^ CD4^+^ T cells stimulated *ex vivo*, following indicated treatments. Each symbol represents an individual mouse (*n* = 9, two independent experiments). Data are shown as mean ± SEM. All *p* values were determined by one-way ANOVA with Tukey’s multiple comparison test except for graphs in (E)–(G) and (N) for which *p* values were determined by unpaired two-tailed Student’s t test. See also Figures S3–S5.

Anti-CTLA-4 antibodies are expected to act primarily on T cells, and their direct activity on blood vessels is unlikely because TA-ECs and TA-HECs do not express mRNAs encoding CTLA-4 and FcγRs (Figure S3A). Accordingly, CD45^−^CD31^high^ tumor endothelial cells and TA-HECs were not modulated in lymphocyte-deficient *Rag2*^−/−^ mice after treatment with 9H10 (Figures S3B and S3C). We previously identified that CD4^+^ T cells, but not CD8^+^ T cells, are required for the development of spontaneous TA-HEVs in untreated MCA_prog_ tumors.^11^ Thus, we first examined the activities of Fc-null and parental 9D9 on tumor-infiltrating CD4^+^ T cells. As reported in other mouse tumor models,^29^ 9D9 reduced the frequency of intratumoral FOXP3^+^ Tregs in an Fc-dependent manner (Figure 2A). The number of FOXP3^−^ conventional CD4^+^ T cells (Tconv) was increased (Figure 2B), while the number of Tregs was not significantly altered (Figure 2C), resulting in a 2-fold increase of the ratio of CD4^+^ Tconv to Tregs in tumors (Figure 2D). In contrast, both Fc-null and parental 9D9 increased Tregs in tumor-draining lymph nodes (TDLNs, Figure S4A), confirming that peripheral expansion of Tregs is Fc-independent during anti-CTLA-4 therapy.^29^ A previous study reported that 9D9 can alter the phenotype of tumor-infiltrating Tregs.^42^ Accordingly, we observed changes in the phenotype of Tregs in MCA_prog_ tumors upon treatment with 9D9. While the expression of FOXP3 did not change, the expression of Tbet, ICOS, PD-1, CTLA-4, and CD39 decreased (Figure 2E). Moreover, the frequency of CD25^+^CTLA-4^+^ Tregs was reduced (Figure 2F). Given that Tbet is an important regulator of Treg stability and function during Th1 immunity,^43^ that ICOS is an important survival factor for tumor-infiltrating Tregs,^44^ and that CD25, CTLA-4, and CD39 are all involved in Treg-mediated immunosuppression,^45^ our results suggested that 9D9 reduces the activation, stability, and function of Tregs in MCA_prog_ tumors.

Since tumor-infiltrating CD4^+^ Tconv were highly expanded during treatment with 9D9, we hypothesized that they could play a role in the modulation of tumor blood vessels. We further examined how they were modulated and focused our analysis on Tbet^+^ICOS^+^ Th1-like CD4^+^ T cells. These non-canonical effector CD4^+^ T cells are induced upon treatment with anti-CTLA-4 antibodies in mice and patients,^22^^,^^24^^,^^25^ due to blockade of the CTLA-4 checkpoint, an important regulator of early CD4^+^ T cell differentiation,^23^ and ICOS-dependent maturation of unskewed Tbet^−^ICOS^+^ CD4^+^ T cells into interferon gamma (IFNγ)-producing Tbet^+^ICOS^+^ Th1-like CD4^+^ T cells.^46^ We found that treatment with 9D9 increased ICOS and Tbet expression in CD4^+^ Tconv (Figures 2G and 2H). Interestingly, CD4^+^ Tconv poorly expressed Tbet in TDLNs (Figure S4B), whereas CD4^+^ Tconv co-expressing ICOS and Tbet were observed in tumors (Figure 2H), suggesting that full engagement of the Th1 program occurs in the tumor microenvironment. While both Fc-null and parental 9D9 increased the frequency and number of Tbet^−^ICOS^+^ Tconv in TDLNs (Figures S4B and S4C), only the parental 9D9 increased the frequency and number of Tbet^+^ICOS^+^ CD4^+^ Tconv in tumors (referred to as Tbet^+^ICOS^+^ Th1-like CD4^+^ T cells in the rest of the manuscript, Figures 2H and 2I), as well as their proliferative (Ki67) activity (Figure 2J). Therefore, the activities of anti-CTLA-4 antibodies on tumor-infiltrating Tbet^+^ICOS^+^ Th1-like CD4^+^ T cells and tumor vasculature are both governed by antibody Fc effector function.

We observed that the numbers of Tbet^+^ICOS^+^ Th1-like CD4^+^ T cells in tumors correlated to reduced frequencies of CD45^−^CD31^high^ tumor endothelial cells and increased frequencies of MECA-79^+^ TA-HECs after anti-CTLA-4 treatment (Figures 2K and 2L). Tbet^+^ICOS^+^ Th1-like CD4^+^ T cells are important producers of IFNγ,^24^ a prototypical Th1 cytokine with anti-angiogenic and vascular-modulating activities.^47^^,^^48^ We found that Tbet^+^ICOS^+^ Th1-like CD4^+^ T cells expressed the highest level of PD-1 among tumor-infiltrating CD4^+^ Tconv (Figure 2M), indicative of high tumor reactivity,^49^ and that *in vivo* treatment with 9D9 increased the proportion of PD-1^+^ CD4^+^ T cells producing IFNγ upon re-stimulation *ex vivo* (Figure 2N). Together, these observations suggested an important role of IFNγ-producing effector CD4^+^ T cells in the vascular remodeling observed during anti-CTLA-4 therapy.

We finally analyzed the impact of 9D9 on intratumoral CD8^+^ T cell phenotypes. We found that 9D9 increased the absolute numbers of tumor-infiltrating CD44^+^PD-1^+^ CD8^+^ T cells, stem-like/progenitor exhausted (SLAMF6^+^TIM3^−^) and effector/terminally exhausted (SLAMF6^−^TIM3^+^) CD8^+^ T cells (Figures S5A–S5C). Moreover, the proliferative activity of stem-like/progenitor exhausted CD8^+^ T cells was increased (Figure S5D), suggesting that 9D9 promotes the expansion and differentiation of stem-like/progenitor exhausted CD8^+^ T cells, as recently described for human anti-CTLA-4 antibodies in patients with metastatic melanoma.^50^

### CD4^+^ T cells are important for the increase of TA-HEVs during anti-CTLA-4 therapy

**Figure 3:**
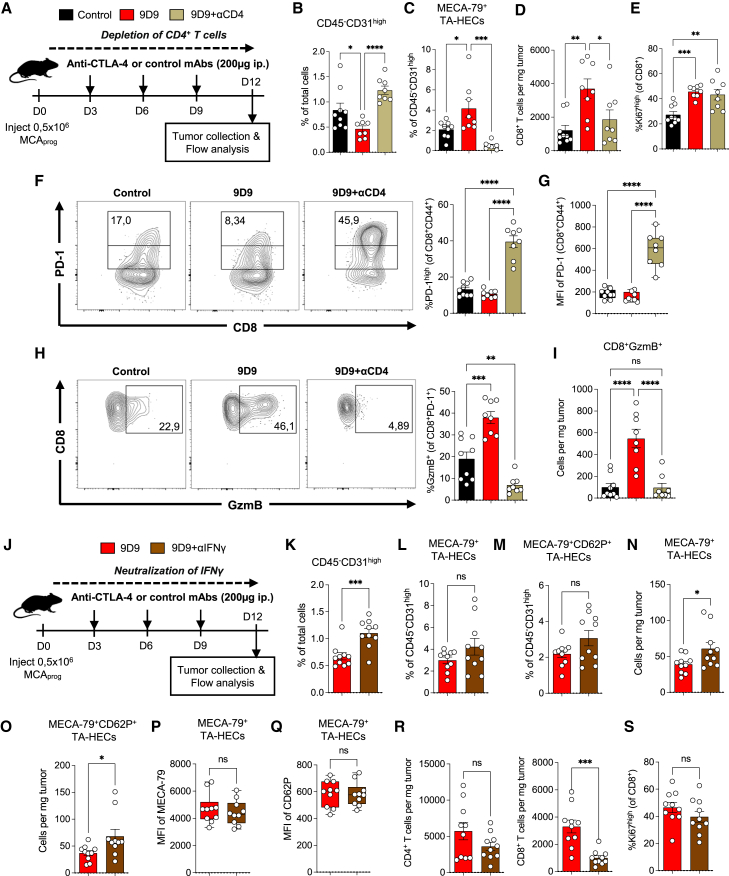
CD4^+^ T cells are important for the increase of TA-HEVs during anti-CTLA-4 therapy (A) Treatment schedule. i.p., intraperitoneal; control, isotype control antibodies. (B and C) Frequencies of CD45^−^CD31^high^ endothelial cells and MECA-79^+^ TA-HECs. Each symbol represents an individual mouse (*n* = 8–9, two independent experiments). (D and E) Number of tumor-infiltrating CD8^+^ T cells and frequency of Ki67^high^ cells in tumor-infiltrating CD8^+^ T cells. Each symbol represents an individual mouse (*n* = 8–9, two independent experiments). (F and G) Frequency of PD-1^high^ cells in tumor-infiltrating CD44^+^ CD8^+^ T cells following indicated treatments, and mean fluorescence intensity (MFI) of PD-1 in the same subset, quantified by flow cytometry. Representative dot plots are shown (F). Each symbol represents an individual mouse (*n* = 8–9, two independent experiments). (H and I) Frequency and number of GzmB^+^ cells in tumor-infiltrating CD44^+^PD-1^+^ CD8^+^ T cells. Representative dot plots are shown (H). Each symbol represents an individual mouse (*n* = 8–9, two independent experiments). (J) Treatment schedule. i.p., intraperitoneal; control, isotype control antibodies. (K–Q) Frequencies of CD45^−^CD31^high^ endothelial cells, TA-HECs, MECA-79^+^CD62P^+^ TA-HECs, number of TA-HECs, and MFI of MECA-79 and CD62P in TA-HECs, following indicated treatments, quantified by flow cytometry. Each symbol represents an individual mouse (*n* = 10, two independent experiments). (R and S) Numbers of tumor-infiltrating CD4^+^ and CD8^+^ T cells, and frequency of Ki67^high^ cells in tumor-infiltrating CD8^+^ T cells. Each symbol represents an individual mouse (*n* = 10, two independent experiments). Data are shown as mean ± SEM. All *p* values were determined by one-way ANOVA with Tukey’s multiple comparison test except for graphs in (K)–(S) for which *p* values were determined by unpaired two-tailed Student’s t test. See also Figures S6 and S7.

We next performed anti-CTLA-4 therapy in tumor-bearing mice depleted of CD4^+^ T cells (Figure 3A). Remarkably, depletion of CD4^+^ T cells largely prevented the anti-angiogenic activity of anti-CTLA-4 antibodies (Figure 3B) and drastically reduced the frequency of MECA-79^+^ TA-HECs (Figure 3C). Additionally, CD4^+^ T cell depletion impaired the accumulation of CD8^+^ T cells in tumors (Figure 3D), which was not due to CD8^+^ T cell-intrinsic proliferative defects (Figure 3E). Finally, CD4^+^ T cell depletion resulted in a strong upregulation of PD-1 on tumor-infiltrating CD8^+^ T cells (Figures 3F and 3G) and defects in the generation of granzyme B^+^ cytotoxic CD8^+^ T cells upon treatment (Figures 3H and 3I). Therefore, CD4^+^ T cells are important for the remodeling of tumor blood vessels and the increase of TA-HEVs, and they also control the recruitment and functional phenotype of CD8^+^ T cells during anti-CTLA-4 therapy.

We then investigated the impact of IFNγ neutralization during anti-CTLA-4 therapy (Figure 3J). IFNγ blockade resulted in increased frequency of CD45^−^CD31^high^ tumor endothelial cells (Figure 3K) but did not alter the frequencies of MECA-79^+^ TA-HECs and bifunctional MECA-79^+^CD62P^+^ TA-HECs (Figures 3L and 3M). In fact, it even resulted in increased numbers of MECA-79^+^ TA-HECs and MECA-79^+^CD62P^+^ TA-HECs (Figures 3N and 3O). In contrast, the MFI of MECA-79 and CD62P on MECA-79^+^ TA-HECs was not affected (Figures 3P and 3Q). Interestingly, IFNγ neutralization limited the infiltration of CD8^+^ T cells into tumors (Figure 3R), which was not due to CD8^+^ T cell-intrinsic proliferative defects (Figure 3S), similar to observations made after CD4^+^ T cell depletion (Figures 3D and 3E). Together, these results indicated that IFNγ is essential for the pruning of CD45^−^CD31^high^ tumor endothelial cells and the accumulation of CD8^+^ T cells into tumors during anti-CTLA-4 therapy but not for the formation of TA-HEVs or the modulation of their phenotype.

We observed that mRNAs encoding the two chains of the IFNγ receptor (IFNGR1 and IFNGR2) were expressed in MECA-79^−^ TA-ECs and MECA-79^+^ TA-HECs (Figure S6A). To investigate whether IFNγ directly acts on tumor endothelial cells, we analyzed the expression of programmed death ligand 1 (PD-L1) as a readout of IFNγ signaling (*Cd274* encoding PD-L1 is an IFNγ-stimulated gene; Figure S6B). MECA-79^+^ TA-HECs expressed high baseline levels of PD-L1 in the absence of treatment, with expression levels largely exceeding those observed in MECA-79^−^ TA-ECs (Figures S6C and S6D). Nonetheless, PD-L1 was increased on MECA-79^+^ TA-HECs during treatment with Fc-competent 9D9 (Figures S6E and S6F), and PD-L1 upregulation was dependent on IFNγ (Figures S6G and S6H), confirming that TA-HEVs are directly affected by IFNγ during treatment. In addition, 9D9 induced a robust Fc-dependent upregulation of PD-L1 on MECA-79^−^ TA-ECs (Figures S7A and S7B), consistent with their potent elimination during treatment (Figure S2D), and this was dependent on both CD4^+^ T cells and IFNγ (Figures S7C and S7D). We concluded that anti-CTLA-4 antibodies prune tumor endothelial cells and upregulate the PD-L1 immune checkpoint on TA-HEVs via Fc- and IFNγ-dependent mechanisms.

### Excessive Fc effector function leads to TA-HEVs pruning during treatment with mouse anti-CTLA-4 antibodies

**Figure 4:**
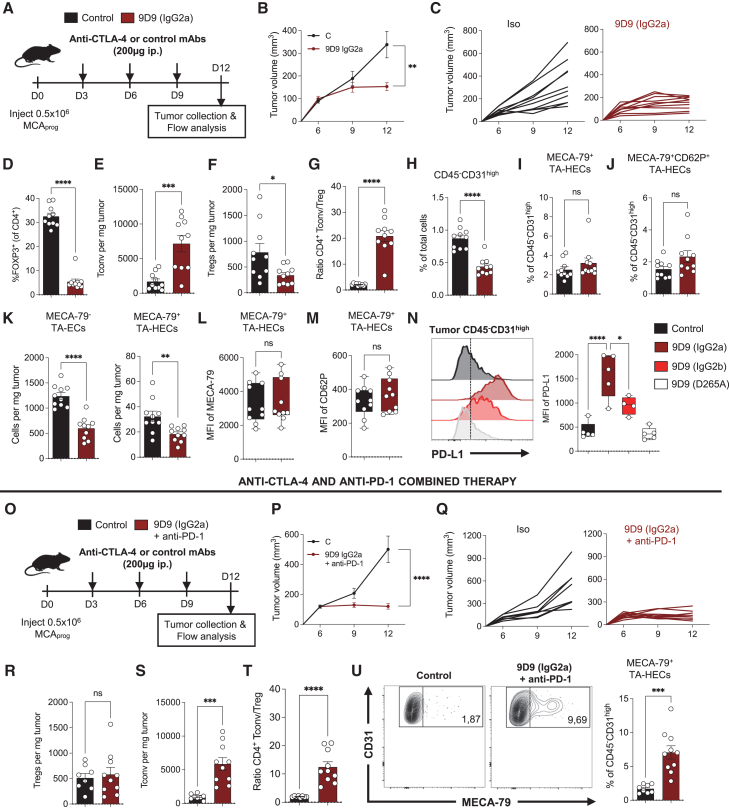
Fc-enhanced mouse anti-CTLA-4 antibodies prune TA-HEVs in the absence of concurrent PD-1 blockade (A) Treatment schedule. i.p., intraperitoneal; control, isotype control antibodies. (B and C) Mean and individual tumor growth. Data were obtained from two independent experiments (*n* = 10 mice per group). (D–G) Frequency of FOXP3^+^ Tregs in tumor, numbers of tumor-infiltrating FOXP3^+^ Tregs and FOXP3^−^ Tconv, and ratio of Tconv to Treg cell numbers in tumors following indicated treatments. Each symbol represents an individual mouse (*n* = 10, two independent experiments). (H–M) Frequencies of CD45^−^CD31^high^ endothelial cells, TA-HECs, MECA-79^+^CD62P^+^ TA-HECs, numbers of TA-ECs and TA-HECs, and mean fluorescence intensity (MFI) of MECA-79 and CD62P in TA-HECs, following indicated treatments, quantified by flow cytometry. Each symbol represents an individual mouse (*n* = 10, two independent experiments). (N) Histograms showing expression of PD-L1 in CD45^−^CD31^high^ tumor endothelial cells following indicated treatments, quantified by flow cytometry. MFI of PD-L1 is quantified. Each symbol represents an individual mouse (*n* = 4–5). (O) Treatment schedule. i.p., intraperitoneal; control, isotype control antibodies. (P and Q) Mean and individual tumor growth. Data were obtained from two independent experiments (control, *n* = 8 mice; 9D9-IgG2a + anti-PD-1, *n* = 10 mice). (R–T) Numbers of tumor-infiltrating FOXP3^+^ Tregs and FOXP3^−^ Tconv, and ratio of Tconv to Tregs cell numbers in tumors following indicated treatments. Each symbol represents an individual mouse (*n* = 8–10, two independent experiments). (U) Frequency of TA-HECs. Representative dot plots of TA-HECs are shown. Each symbol represents an individual mouse (*n* = 8–10, two independent experiments). Data are shown as mean ± SEM. All *p* values were determined by unpaired two-tailed Student’s t test except for graph in (N) for which *p* values were determined by one-way ANOVA with Tukey’s multiple comparison test, and for (B) and (P) for which *p* values were determined by two-way ANOVA. See also Figures S8–S10.

Since the increase of TA-HEVs relies on Fc-dependent mechanisms during anti-CTLA-4 therapy, we next examined how an Fc-enhanced version of 9D9 (mouse IgG2a Fc, Figure S8A) would modulate tumor-infiltrating CD4^+^ T cells and TA-HEVs. We found that Fc-enhanced 9D9 improved tumor control (Figures 4A–4C) and drastically reduced the frequency of Tregs in tumors (Figure 4D). The number of intratumoral CD4^+^ Tconv was increased (Figure 4E), and the number of intratumoral Tregs was significantly reduced (Figure 4F), resulting in a 10-fold increase of the ratio of CD4^+^ Tconv to Tregs in tumors (Figure 4G). We then examined the impact of the treatment on tumor endothelial cells. Fc-enhanced 9D9 had a potent anti-angiogenic activity (Figure 4H) but did not increase the frequencies of MECA-79^+^ and MECA-79^+^CD62P^+^ TA-HECs (Figures 4I and 4J). In fact, it significantly reduced the numbers of both MECA-79^−^ TA-ECs and MECA-79^+^ TA-HECs (Figure 4K), with no changes in TA-HEC phenotype (Figures 4L and 4M). Fc-enhanced 9D9 also induced higher levels of endothelial PD-L1, as compared to Fc-null and parental 9D9 mAbs (Figure 4N), revealing increased exposure of tumor endothelial cells to IFNγ. We concluded that excessive Fc effector function leads to detrimental effects on TA-HEVs during treatment with mouse anti-CTLA-4 antibodies.

Additional analyses at an earlier time point (day 8, Figure S8B) revealed that treatment with two cycles of Fc-enhanced 9D9 was sufficient to elicit anti-angiogenic effect (Figure S8C). In this setting, Fc-enhanced 9D9 also reduced the numbers of both MECA-79^−^ TA-ECs and MECA-79^+^ TA-HECs (Figure S8D) and did not modify the frequency of MECA-79^+^ TA-HECs (Figure S8E). Moreover, it upregulated endothelial PD-L1 (Figure S8F), decreased the frequency and number of intratumoral Tregs (Figures S8G and S8H), and increased the proportion of IFNγ-producing PD-1^+^ CD4^+^ T cells (Figure S8I). In contrast, at this early time point, the parental 9D9 had no significant effects on tumor blood vessels, PD-L1 expression, intratumoral Tregs, and frequency of IFNγ-producing CD4^+^ T cells (Figures S8C–S8I). These experiments indicated that the Fc-enhanced 9D9, which depletes Tregs, had more potent anti-angiogenic effects than the parental 9D9 mAb.

In another series of experiments, we found that treatment with anti-CD25 mAb PC-61, which reduced the absolute number of intratumoral Tregs without concomitant activation of CD4^+^ Tconv (Figures S9A–S9E), did not have any impact on CD45^−^CD31^high^ tumor endothelial cells, MECA-79^−^ TA-ECs, and MECA-79^+^ TA-HECs (Figures S9F–S9H). These observations suggested that activation of effector CD4^+^ T cells is crucial for the remodeling of tumor blood vessels in the MCA_prog_ tumor model. Collectively, our data indicate that the vascular-modulating properties of mouse anti-CTLA-4 antibodies are tightly regulated by their Fc effector function and the associated balance of CD4^+^ T conv and Tregs in tumors.

### Concomitant PD-1 blockade rescues the increase of TA-HEVs during treatment with Fc-enhanced mouse anti-CTLA-4 antibodies

Recent studies revealed that cell-intrinsic expression of PD-1 limits Treg pools,^51^^,^^52^ resulting in their expansion during PD-1/PD-L1 blockade. We thus hypothesized that combining the Fc-enhanced 9D9 IgG2a mAb with PD-1 blockade could mitigate the reduction of intratumoral Tregs and, as such, rescue the increase of TA-HEVs. Importantly, we first confirmed that treatment with anti-PD-1 mAb RMP1-14 alone had no impact on tumor growth and tumor endothelial cells in the MCA_prog_ tumor model (Figures S9I–S9M).^11^ Then, we found that combined therapy with Fc-enhanced 9D9 and anti-PD-1 mAbs was associated with potent antitumor activity (Figures 4O and 4P), with multiple tumors displaying a regressing trajectory (Figure 4Q). The frequency of Tregs was reduced in tumors (Figure S10A), but not their absolute number (Figure 4R), indicating that PD-1 blockade limits the reduction of intratumoral Tregs induced by Fc-enhanced 9D9. However, the number of intratumoral CD4^+^ Tconv was still significantly increased (Figure 4S), leading to a 6-fold increase in the ratio of CD4^+^ Tconv to Tregs in tumors (Figure 4T). Remarkably, the combined therapy maintained anti-angiogenic activity (Figure S10B) and highly increased the frequency of MECA-79^+^ TA-HECs (Figure 4U). The number of MECA-79^−^ TA-ECs was reduced (Figure S10C) but not the number of MECA-79^+^ TA-HECs that was almost significantly increased (*p* = 0.0666, Figure S10D). Furthermore, Fc-enhanced and parental 9D9 induced similar upregulation of endothelial PD-L1 when combined with PD-1 blockade (Figure S10E). Finally, we observed that combined therapy elicited potent CD8^+^ T cell antitumor immunity (Figures S10F and S10G). Collectively, our results indicated that Fc-enhanced mouse anti-CTLA-4 antibodies increase TA-HEVs and improve antitumor immunity in combination with anti-PD-1 antibodies.

### Ipilimumab requires increased Fc effector function to modulate TA-HEVs in humanized CTLA-4/FcγR mice

**Figure 5:**
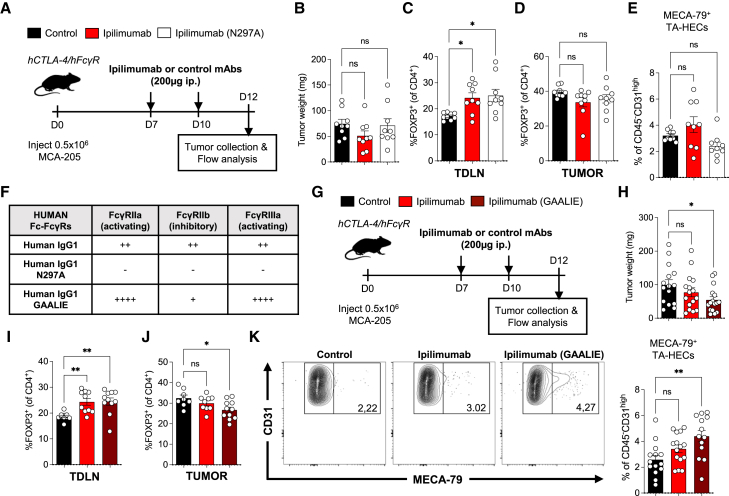
The human anti-CTLA-4 antibody ipilimumab requires increased Fc effector function to modulate TA-HEVs in humanized mice (A) Treatment schedule. i.p., intraperitoneal; control, isotype control antibodies. (B) Tumor weights in individual mice. Each symbol represents an individual mouse (*n* = 9, two independent experiments). (C and D) Frequencies of FOXP3^+^ Tregs in tumor-draining lymph nodes (TDLNs) and tumors following indicated treatments. Each symbol represents an individual mouse (*n* = 9, two independent experiments). (E) Frequency of TA-HECs. Each symbol represents an individual mouse (*n* = 9, two independent experiments). (F) Table presenting the binding profiles to human FcγRs of the various IgGs used in the study. Relative binding affinities were defined based on affinity constants previously assessed by surface plasmon resonance or other binding assays.^57^^,^^58^ (G) Treatment schedule. i.p., intraperitoneal; control, isotype control antibodies. (H) Tumor weights in individual mice. Each symbol represents an individual mouse (*n* = 15–16, three independent experiments). (I and J) Frequencies of FOXP3^+^ Tregs in TDLNs and tumors following indicated treatments. Each symbol represents an individual mouse (*n* = 8–11, two independent experiments). (K) Frequency of TA-HECs. Representative dot plots of TA-HECs are shown. Each symbol represents an individual mouse (*n* = 13–15, three independent experiments). Data are shown as mean ± SEM. All *p* values were determined by one-way ANOVA with Tukey’s multiple comparison test. See also Figure S11.

Mouse and human FcγR systems significantly differ,^53^ and although the role of Fc effector function in the antitumor activity of mouse anti-CTLA-4 antibodies has been clearly demonstrated in mouse tumor models,^27^^,^^28^^,^^29^^,^^30^^,^^31^^,^^32^^,^^37^ its importance in patients is controversial,^34^ especially because human anti-CTLA-4 antibodies fail to induce Fc-dependent depletion of intratumoral Tregs.^33^ To determine whether the vascular-modulating properties of anti-CTLA-4 antibodies are conserved in the human setting, we took advantage of a humanized mouse model expressing both human CTLA-4 and human FcγRs instead of the mouse protein equivalents (Figures S11A–S11C).^35^ These mice, referred to as “humanized CTLA-4/FcγR mice,” were obtained by crossing human CTLA-4 transgenic mice with humanized FcγR mice,^54^^,^^55^ providing an immunocompetent mouse model for *in vivo* evaluation of fully human anti-CTLA-4 antibodies. We generated recombinant antibodies composed of the variable heavy- and light-chain sequences of ipilimumab, the main human anti-CTLA-4 antibody used in patients, with either its original human IgG1 Fc (exactly mimicking the clinical product) or the Fc-null human IgG1 variant N297A that does not bind human FcγRs.^56^ When tested in the MCA-205 tumor model (Figure 5A), none of these antibodies altered tumor growth (Figure 5B), yet both antibodies increased Tregs in TDLNs (Figure 5C), confirming their checkpoint-blocking activity *in vivo*. In addition, ipilimumab failed to reduce tumor-infiltrating Tregs (Figure 5D) and did not significantly increase MECA-79^+^ TA-HECs despite a trend for higher frequency that was not observed with Fc-null ipilimumab (Figure 5E). These results indicated that ipilimumab is biologically active in humanized CTLA-4/FcγR mice, but its antitumor activity and capacity to modulate TA-HEVs appear limited, which could be due to its modest Fc effector function.^35^

We hypothesized that increasing the Fc effector function of ipilimumab could unlock its capacity to modulate TA-HEVs. We thus produced an Fc-engineered version of ipilimumab containing the human IgG1 Fc variant GAALIE.^57^^,^^58^ This Fc variant has increased binding to human activating FcγRs and reduced binding to the human inhibitory FcγRIIb (Figure 5F). This latter property is particularly important as FcγRIIb is highly expressed in the tumor microenvironment where it limits the Fc-dependent activity of human IgG antibodies.^35^ We evaluated Fc-optimized ipilimumab (GAALIE) in comparison with the original ipilimumab in the MCA-205 tumor model (Figure 5G). We found that Fc engineering improved the antitumor activity of ipilimumab (Figure 5H). Although both antibodies increased Tregs in TDLNs (Figure 5I), only the Fc-optimized ipilimumab reduced Tregs in tumors (Figure 5J) and significantly increased the frequency of MECA-79^+^ TA-HECs (Figure 5K). Collectively, these results showed that ipilimumab requires increased Fc effector function to modulate TA-HEVs in humanized CTLA-4/FcγR mice.

### Fc-optimized ipilimumab combined with PD-1 blockade increases TA-HEVs and melanoma tumor control

**Figure 6:**
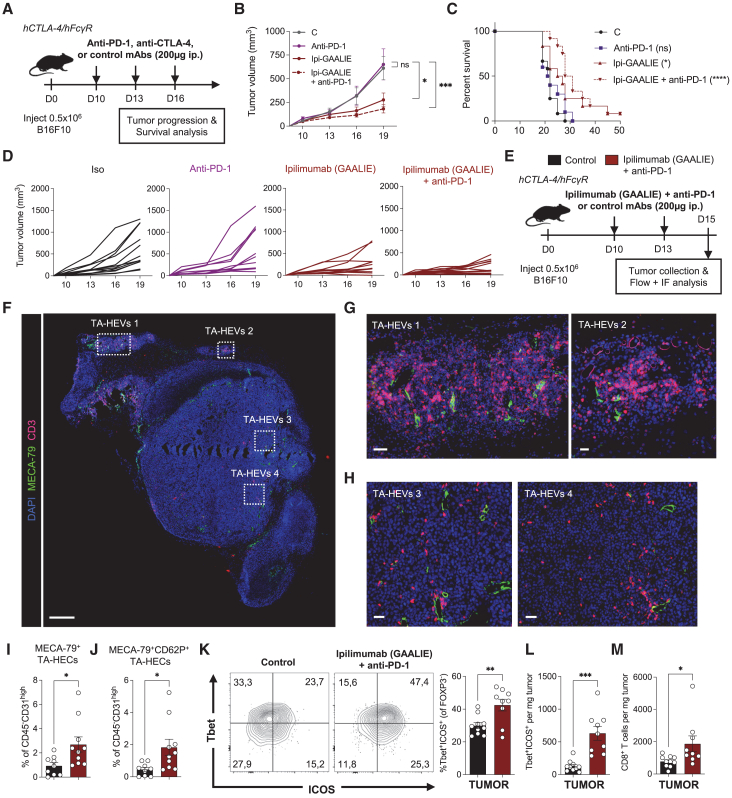
Fc-optimized ipilimumab combined with anti-PD-1 increases TA-HEVs and melanoma tumor control in humanized mice (A) Treatment schedule. i.p., intraperitoneal; control, isotype control antibodies. (B–D) Mean tumor growth, Kaplan-Meier survival curves, and individual tumor growth. Data were obtained from two independent experiments (control, *n* = 12 mice; anti-PD-1, *n* = 10 mice; ipilimumab-GAALIE, *n* = 12 mice; ipilimumab-GAALIE + anti-PD-1, *n* = 12 mice). (E) Treatment schedule. i.p., intraperitoneal; IF, immunofluorescence; control, isotype control antibodies. (F–H) Immunofluorescence of B16F10 tumor (day 15) following treatment with ipilimumab-GAALIE + anti-PD-1 antibodies. Selected markers are presented. Inset in (G) and (H) show higher magnifications of boxed areas. Scale bars: 500 μm (F), 50 μm (G left, H left, and H right), 20 μm (G right). (I and J) Frequencies of TA-HECs and MECA-79^+^CD62P^+^ TA-HECs. Each symbol represents an individual mouse (*n* = 9–10, two independent experiments). (K and L) Frequency and numbers of Tbet^+^ICOS^+^ Th1-like CD4^+^ T cells in tumors. Representative dot plots are shown (K). Each symbol represents an individual mouse (*n* = 9–10, two independent experiments). (M) Number of tumor-infiltrating CD8^+^ T cells. Each symbol represents an individual mouse (*n* = 9–10, two independent experiments). Data are shown as mean ± SEM. All *p* values were determined by unpaired two-tailed Student’s t test except for (B) for which *p* values were determined by two-way ANOVA with Dunnett’s multiple comparison test, and for graph in (C) for which *p* values were determined by log rank test.

We previously observed that Fc-optimized ipilimumab (GAALIE), but not original ipilimumab, had antitumor activity in the B16F10 melanoma model,^35^ a poorly infiltrated cold tumor model that is not responsive to ICT.^59^ We tested the Fc-optimized ipilimumab in combination with anti-PD-1 antibodies in this aggressive melanoma model (Figure 6A), mimicking one of the main indications of anti-CTLA-4 antibodies in patients with cancer.^1^^,^^2^ While anti-PD-1 mAb RMP1-14 did not show antitumor activity (Figures 6B–6D), Fc-optimized ipilimumab significantly reduced tumor growth, and its combination with PD-1 blockade further improved tumor control (Figures 6B–6D). Moreover, the combination therapy provided the best survival benefits (Figure 6C). Collectively, these results indicated that Fc-optimized ipilimumab in combination with PD-1 blockade limits tumor growth in the aggressive B16F10 cold tumor model.

We next determined whether TA-HEV modulation is observed in B16F10 melanoma tumors during combined therapy with Fc-optimized ipilimumab and anti-PD-1 antibodies (Figure 6E). Immunostaining of B16F10 melanoma sections revealed the presence of multiple MECA-79^+^ TA-HEVs in CD3^+^ T cell-rich tumor areas after treatment (Figures 6F–6H). MECA-79^+^ TA-HEVs were observed in the tumor periphery close to prominent CD3^+^ T cell infiltrates (Figure 6G), and deeper in the tumor parenchyma close to less dense CD3^+^ T cell infiltrates (Figure 6H). Flow cytometry analysis further revealed that combined therapy significantly increased the frequency of MECA-79^+^ TA-HECs and MECA-79^+^CD62P^+^ TA-HECs (Figures 6I and 6J) and tumor infiltration of Tbet^+^ICOS^+^ Th1-like CD4^+^ T cells and CD8^+^ T cells (Figures 6K–6M). Together, these results demonstrated the capacity of the combined therapy to increase TA-HEVs in a cold tumor model in which TA-HEVs are barely observed at baseline.^60^ Overall, our findings indicated that the human anti-CTLA-4 antibody ipilimumab can modulate TA-HEVs when adequately Fc optimized, revealing a promising avenue for the therapeutic modulation of TA-HEVs in patients with cancer.

## Discussion

Our present findings demonstrate that anti-CTLA-4 antibodies remodel tumor vasculature through Fc-dependent mechanisms. Fc-optimized anti-CTLA-4 antibodies induce important quantitative and qualitative changes in tumor blood vessels that culminate in the increase of TA-HEVs, specialized blood vessels supporting lymphocyte entry into tumors, resulting in improved CD4^+^ and CD8^+^ T cell infiltration into tumors and better response to PD-1 blockade in both conventional and humanized mouse models. These results underscore the ability of Fc-optimized anti-CTLA-4 antibodies to stimulate antitumor immunity through the modulation of TA-HEVs, which could represent a clinically relevant opportunity for cold tumors that are poorly infiltrated by T cells and that show poor response to ICT.^3^^,^^4^

Our results identify an intricate interplay between the Fc effector function of anti-CTLA-4 antibodies, IFNγ-producing effector CD4^+^ T cells, and tumor endothelial cells. Anti-CTLA-4 antibodies with optimal Fc effector function reinforce the MECA-79^+^CD62P^+^ bifunctional phenotype of TA-HECs and increase their proportion in tumor microvasculature by selectively pruning MECA-79^−^ non-HEV TA-ECs, while Fc-null anti-CTLA-4 antibodies do not have any activity on tumor endothelial cells. The remodeling of tumor blood vessels and anti-angiogenic effects of anti-CTLA-4 antibodies require the presence of CD4^+^ T cells and the activity of IFNγ, a potent anti-angiogenic cytokine.^47^^,^^48^ ICT has previously been shown to increase tumor blood vessel normalization and perfusion.^61^^,^^62^ Thus, we believe that pruning of dysfunctional non-HEV tumor blood vessels upon anti-CTLA-4 treatment, resulting from the anti-angiogenic effects of IFNγ, will lead to an increased functionality and a better perfusion of the remaining vessels, including TA-HEVs. Importantly, the tumor vasculature will also divide into a lower number of branches. As a result, more recirculating lymphocytes will enter TA-HEV branches in the tumor microcirculation and will thus interact with TA-HECs (Figure S12A), which will likely result in increased numbers of lymphocytes that roll, stick, and extravasate through TA-HEVs. The quality of TA-HECs and their degree of functionality are also important for lymphocyte capture and immigration through TA-HEVs,^11^ as observed for lymph node HEVs.^15^ MECA-79^+^ TA-HEVs co-express MECA-79 ligands (for lymphocyte CD62L) and CD62P receptor (for lymphocyte PSGL-1), and these two adhesion pathways are crucial for the capture and rolling of lymphocytes in TA-HEVs and the increased infiltration of CD4^+^ and CD8^+^ T cells into tumors during ICT.^11^ We found that anti-CTLA-4 antibodies increased both MECA-79 MFI and CD62P MFI on MECA-79^+^ TA-HECs in an Fc-dependent manner. Upregulation of MECA-79 and CD62P on TA-HECs is thus also likely to contribute to increased lymphocyte capture and extravasation into tumors during treatment (Figure S12B).

We observed that Fc-enhanced mouse anti-CTLA-4 antibodies (9D9 mouse IgG2a) eliminate tumor endothelial cells indiscriminately, including the beneficial MECA-79^+^ TA-HECs. Our results suggest that a strong depletion of Tregs combined to prominent expansion of effector T cells could lead to excessive IFNγ-dependent responses in the tumor microenvironment, which may explain the unrestrained pruning of tumor endothelial cells in this setting. This has important clinical implications as TA-HEVs are major sites of lymphocyte entry into tumors during ICT.^11^ Furthermore, while IFNγ is critical for successful response to ICT,^6^^,^^63^ intense and prolonged IFNγ signaling is also associated to defective antitumor immunity and acquired resistance,^64^^,^^65^ suggesting that IFNγ-dependent responses should respect a certain threshold to remain beneficial during anti-CTLA-4 therapy. Interestingly, we found that combined PD-1 blockade could rescue TA-HEVs increase during treatment with Fc-enhanced mouse anti-CTLA-4 antibodies, which we propose to be due to the restoration of an optimal balance between effector T cells and Tregs in tumors. On the other hand, our studies in humanized CTLA-4/FcγR mice revealed that Fc-enhanced human anti-CTLA-4 antibodies (ipilimumab IgG1 GAALIE) do not behave as their murine counterparts since they increase TA-HEVs even when they are used alone. However, our results still raise concerns about the judicious evaluation of Fc-enhanced anti-CTLA-4 antibodies in patients,^66^ and we believe that they should be exclusively administered in combination with PD-1 blockade to avoid potential deleterious effects in the tumor microenvironment and ensure maximal therapeutic efficacy.

Our data suggest an important contribution of Tbet^+^ICOS^+^ Th1-like CD4^+^ T cells in the remodeling of tumor blood vessels and the increase of TA-HEVs during treatment with anti-CTLA-4 antibodies. Tbet^+^ICOS^+^ Th1-like CD4^+^ T cells are induced upon CTLA-4 blockade in both mice and patients,^22^^,^^24^^,^^25^ and similar cells have been described in *Ctla4*^−/−^ mice,^67^ suggesting that disruption of CTLA-4 checkpoint is critical for their induction. Interestingly, we found that engagement of FcγRs by anti-CTLA-4 antibodies is required for upregulation of the Th1-associated transcription factor Tbet in tumor-infiltrating effector CD4^+^ T cells, suggesting that factors derived from FcγR-expressing cells could be important for the maturation and function of Tbet^+^ICOS^+^ Th1-like CD4^+^ T cells. The cellular crosstalk and the key factors involved remain to be defined, but these interactions appear critical for the remodeling of tumor vasculature and the increase of TA-HEVs during treatment. Interestingly, we observed that anti-PD-1 antibodies fail to remodel tumor vasculature and increase TA-HEVs in our mouse preclinical model. Previous studies established that anti-CTLA-4 and anti-PD-1 therapies act through distinct cellular mechanisms, with one important difference being the incapacity of anti-PD-1 antibodies to induce Tbet^+^ICOS^+^ Th1-like CD4^+^ T cells.^22^ We believe that lack of this effector CD4^+^ T cell population could underlie the absence of vascular remodeling during anti-PD-1 therapy. In agreement with previous observations,^42^ we also observed changes in the phenotype of tumor-infiltrating Tregs upon treatment with anti-CTLA-4 mAb 9D9, suggesting that anti-CTLA-4 therapy reduces the activation, stability, and function of Tregs. Moreover, 9D9 mAb increased the proportion and absolute number of CD8^+^ T cells producing granzyme B. Thus, reduced Treg function and increased CD8^+^ T cell effector function could also contribute to the remodeling of the tumor vasculature and the increase of TA-HEVs during anti-CTLA-4 therapy. Collectively, our results align with previous findings showing that tumor blood vessels and tumor-infiltrating T cells are engaged in a mutual regulatory loop,^61^^,^^62^ with the former regulating the infiltration of T cells into tumors and the latter regulating the abundance and phenotype of tumor blood vessels. In this study, we further defined the mechanisms by which T cells modulate tumor vasculature and TA-HEVs, and we identified how antibody Fc effector function regulates this activity in the setting of anti-CTLA-4 therapy, informing therapeutic opportunities for the modulation of tumor blood vessels and TA-HEVs in patients with cancer.

Surprisingly, we detected higher expression levels of the PD-L1 immune checkpoint on MECA-79^+^ TA-HECs than on MECA-79^−^ TA-ECs, both at baseline and after anti-CTLA-4 therapy. This result was unexpected since TA-HEVs are mainly located in the tumor periphery^9^^,^^11^ and, as such, should be distant from T cell effector responses that may occur deeper in the tumor parenchyma. However, IFNγ diffuses in the tumor microenvironment and can reach considerable distance from its initial site of release,^68^^,^^69^ which may explain why TA-HEVs are also affected by this cytokine. The higher expression levels of PD-L1 on TA-HECs are also likely to be explained by post-transcriptional mechanisms. Indeed, glycosylation of PD-L1 by the core-1 glycosyltransferase B3GNT3 increases its expression levels and controls its interaction with PD-1.^70^^,^^71^ This B3GNT3 enzyme is the critical glycosyltransferase generating the HEV-specific MECA-79 epitope^72^ and, as such, is highly expressed in MECA-79^+^ TA-HECs.^11^ Therefore, B3GNT3 is likely to glycosylate and stabilize PD-L1 in TA-HEVs, resulting in increased expression levels of PD-L1 at the cell surface. Importantly, PD-L1 on TA-HEVs could serve as a primary inhibitory checkpoint for lymphocytes shortly after their extravasation through TA-HEVs, which provides an additional rationale for the use of anti-CTLA-4 antibodies, including Fc-enhanced anti-CTLA-4 antibodies, in combination with PD-1/PD-L1 blockade.

Considering our present findings and past results, Fc-optimized anti-CTLA-4 antibodies appear as multifunctional drugs that could promote antitumor immunity through multiple arms. These activities include (1) blockade of the CTLA-4 checkpoint, resulting in the induction of non-canonical CD4^+^ T cell subsets (i.e., Tbet^+^ICOS^+^ Th1-like CD4^+^ T cells)^22^ and the priming of novel CD8^+^ T cell clonotypes in the periphery,^21^ (2) Fc-dependent depletion of FOXP3^+^ Tregs in tumors^27^ and inhibition of their immunosuppressive function,^42^ and (3) Fc-dependent remodeling of tumor blood vessels and increase of TA-HEVs. Interestingly, these effects could be all synergistic as Treg depletion or inhibition will facilitate the activity of intratumoral effector T cells, and the increase of TA-HEVs will improve the recruitment of peripheral T cells into tumors. Using a humanized mouse model, we found that the first-generation human anti-CTLA-4 antibody ipilimumab does not modulate TA-HEVs because of its limited Fc effector function. This suggests that beneficial Fc-dependent effects are likely to be missed by first-generation anti-CTLA-4 antibodies in patients and that Fc-optimized anti-CTLA-4 antibodies could potentially improve treatment efficacy. In line with this notion, botensilimab, a second-generation Fc-enhanced anti-CTLA-4 antibody, has recently shown encouraging clinical activity in combination with PD-1 blockade in relapsed/refractory microsatellite stable metastatic colorectal cancer^73^ and relapsed/refractory metastatic sarcomas,^74^ two hard-to-treat diseases generally unresponsive to ICT. In future studies, it will be important to determine whether botensilimab increases TA-HEVs and T cell infiltration in these poorly immunogenic/cold tumors.

In conclusion, we demonstrate that anti-CTLA-4 antibodies remodel tumor blood vessels and increase TA-HEVs via Fc-dependent mechanisms, and we identify Fc-optimized anti-CTLA-4 antibodies as effective and clinically applicable therapeutic agents for increasing TA-HEVs and T cell antitumor immunity in patients with cancer.

### Limitations of the study

We found that anti-CTLA-4 antibodies remodel tumor vasculature and increase TA-HEVs through Fc-dependent mechanisms. While this activity was not shared by anti-PD-1 antibodies in our studies, anti-PD-1/PD-L1 antibodies, especially when combined with other therapies, were shown to modulate TA-HEVs in other tumor models.^18^^,^^75^ Furthermore, we found that effector CD4^+^ T cells, particularly Tbet^+^ICOS^+^ Th1-like CD4^+^ T cells, were important for the remodeling of tumor vasculature and the increase of TA-HEVs during anti-CTLA-4 therapy in the MCA_prog_ tumor model. However, we believe that other immune cell populations, such as CD8^+^ T cells and natural killer cells,^12^^,^^60^ could elicit similar activities in other tumor models. Finally, our studies mainly relied on flow cytometry and immunofluorescence analysis. It will be interesting in future studies to perform single-cell RNA sequencing (scRNA-seq) analysis to further characterize the molecular mechanisms involved in the remodeling of tumor endothelial cells during anti-CTLA-4 therapy.

## Resource availability

### Lead contact

Further information and requests for resources and reagents should be directed to and will be fulfilled by the lead contact, Jean-Philippe Girard (jean-philippe.girard@ipbs.fr).

### Materials availability

Requests concerning recombinant antibodies or mouse strains should be directed to the corresponding author Lucas Blanchard (lblanchard@rockefeller.edu) or Jeffrey V. Ravetch (ravetch@rockefeller.edu).

### Data and code availability

• scRNA-seq raw data have been deposited in NCBI’s Gene Expression Omnibus (GEO) and are publicly available. Accession number is listed in the deposited data section of the key resources table.
• All other data reported in this manuscript are available from the lead contact upon request.
• This study does not report original code.
• Any additional information required to reanalyze the data reported in this work paper is available from the lead contact upon request.

## Acknowledgments

We thank all the members of the Girard and Ravetch labs for helpful feedback and discussions. We would like to thank P. Smith, B. Bhagwandin-Colisi, A. Martin Mozqueda, E. Lam, and R. Peraza for maintaining the humanized mouse strains in the Ravetch lab. We are grateful to IPBS ANEXPLO and TRI-Cytometry platforms for excellent technical assistance. We acknowledge the help of E. Näser and A. Métais for flow cytometry and immunofluorescence experiments. We thank R. Schreiber for providing the MCA_prog_ tumor cell line. This work was supported by grants from Ligue Nationale Contre le Cancer (Equipe Labellisée LIGUE 2023 to J.-P.G.), Institut National du Cancer (INCA_16738 and INCa_2017-155 to J.-P.G.), Inserm Cancer (to J.-P.G.), Fondation ARC pour la Recherche sur le Cancer (PGA1 RF20180206911 and “Grand Prix Oberling-Haguenau” to J.-P.G.), Bristol Myers Squibb Foundation for Research in Immuno-Oncology (to J.-P.G.), and Laboratoire d’Excellence Toulouse Cancer (LABEX TOUCAN, ANR-11-LABX-0068). L.B., E.V., and J.L. were supported by fellowships from 10.13039/501100002915Fondation pour la Recherche Médicale (ECO201806006827, ECO202006011469, FDT202106012889, FDT202304016914, and FDT202404018574). Research reported in this publication was also supported by the 10.13039/100000054National Cancer Institute at the National Institutes of Health under award numbers R01CA244327 and R35CA196620 (to J.V.R.). The content is solely the responsibility of the authors and does not necessarily represent the official views of the National Institutes of Health. Schematics were created with BioRender.com.

## Author contributions

L.B. and J.-P.G. conceived the study, designed the experiments, analyzed the data, and wrote the manuscript. L.B. and E.V. performed the experiments and acquired data. D.A.K. contributed to experiment design and data analysis. J.V.R. provided critical resources, contributed to experiment design, and data analysis. J.L., C.M., D.T., and N.O. assisted during flow cytometry and immunofluorescence experiments. M.B. and A.M. produced recombinant antibodies. J.-P.G. supervised the work.

## Declaration of interests

J.V.R. is an inventor on a patent (WO2019125846A1) describing the GAALIE variant and its use for therapeutic monoclonal antibodies.

## STAR★Methods

### Key resources table

**Table:** 

REAGENT or RESOURCE	SOURCE	IDENTIFIER
**Antibodies**		

Rat anti-Mouse High Endothelial Venule Hybridoma Supernatant (Clone MECA-79)	ATCC	Cat# ATCC HB-9479; RRID: CVCL_9222
Hamster anti-Mouse CD3e (Clone 145-2C11)	BD Biosciences	Cat# 557306; RRID: AB_394591
Rat anti-Mouse CD31 (Clone MEC 13.3)	BD Biosciences	Cat# 550274; RRID: AB_393571
Rat anti-Mouse CD62P-AF647 (Clone RB40.34)	BD Biosciences	Cat# 563674; RRID: AB_2738366
Rat anti-Mouse IFNγ-AF647 (Clone XMG1.2)	BD Biosciences	Cat# 557735; RRID: AB_396843
Rat anti-Mouse High Endothelial Venule-biotin (Clone MECA-79)	BioLegend	Cat# 120804; RRID: AB_493557
Rat anti-Mouse CD11b-BV711 (Clone M1/70)	BioLegend	Cat# 101241; RRID: AB_11218791
Rat anti-Mouse CD3e-PE-Cy7 (Clone 17A2)	BioLegend	Cat# 100219; RRID: AB_1732068
Rat anti-Mouse CD4-AF488 (Clone GK1.5)	BioLegend	Cat# 100425; RRID: AB_493520
Rat anti-Mouse CD8a-PB (Clone 53–6.7)	BioLegend	Cat# 100725; RRID: AB_493425
Rat anti-Mouse CD8a-APC-Cy7 (Clone 53–6.7)	BioLegend	Cat# 100713; RRID: AB_312752
Rat anti-Mouse CD8a-FITC (Clone 53–6.7)	BioLegend	Cat# 100705; RRID: AB_312744
Mouse anti-Human CD16/FcγRIII-PE-Cy7 (Clone 3G8)	BioLegend	Cat# 302015; RRID: AB_314215
Rat anti-Mouse CD31-PB (Clone 390)	BioLegend	Cat# 102422; RRID: AB_10613457
Rat anti-Mouse CD44-APC-Cy7 (Clone IM7)	BioLegend	Cat# 103027; RRID: AB_830784
Rat anti-Mouse CD45-PerCP (Clone 30-F11)	BioLegend	Cat# 103130; RRID: AB_893339
Mouse anti-Human CD64/FcγRI-PE (Clone 10.1)	BioLegend	Cat# 305007; RRID: AB_314491
Hamster anti-Mouse CD152/CTLA-4-BV421 (Clone UC10-4B9)	BioLegend	Cat# 106311; RRID: AB_10901170
Rat anti-Mouse CD25-PE (Clone PC61)	BioLegend	Cat# 102007; RRID: AB_312856
Mouse anti-Human CD152/CTLA-4-PE (Clone L3D10)	BioLegend	Cat# 349905; RRID: AB_10645522
Rat anti-Mouse CD278/ICOS-BV711 (Clone C398.4A)	BioLegend	Cat# 313547; RRID: AB_2734288
Rat anti-Mouse CD39-PE-Cy7 (Clone Duha59)	BioLegend	Cat# 143805; RRID: AB_2563393
Mouse anti-Mouse granzyme B-PB (Clone GB11)	BioLegend	Cat# 515407; RRID: AB_2562195
Rat anti-Mouse Ki67-PB (Clone 16A8)	BioLegend	Cat# 652421; RRID: AB_2564489
Rat anti-Mouse PD-1-PE-Cy7 (Clone 29F.1A12)	BioLegend	Cat# 135215; RRID: AB_10696422
Rat anti-Mouse PD-L1-PE-Cy7 (Clone 10F.9G2)	BioLegend	Cat# 124313; RRID: AB_10643573
*InVivo*MAb Rat anti-Mouse CD4 (clone GK1.5)	Bio X Cell	Cat# BE0003-1; RRID: AB_1107636
*InVivo*MAb Mouse anti-Mouse CTLA-4 (clone 9D9)	Bio X Cell	Cat# BE0164; RRID: AB_10949609
*InVivo*MAb Syrian Hamster anti-Mouse CTLA-4 (clone 9H10)	Bio X Cell	Cat# BE0131; RRID: AB_10950184
RecombiMAb Mouse IgG1 anti-Mouse CTLA-4 (clone 9H10-CP146)	Bio X Cell	Cat# CP146; RRID: AB_2927521
*InVivo*MAb Rat anti-Mouse IFNγ (clone XMG1.2)	Bio X Cell	Cat# BE0055; RRID: AB_1107694
*InVivo*MAb Rat anti-Mouse PD-1 (clone RMP1-14)	Bio X Cell	Cat# BE0146; RRID: AB_10949053
*InVivo*MAb Human IgG1 isotype control	Bio X Cell	Cat# BE0297; RRID: AB_2687817
*InVivo*MAb Mouse IgG2b isotype control (clone MPC-11)	Bio X Cell	Cat# BE0086; RRID: AB_1107791
*InVivo*MAb Rat IgG1 isotype control (clone HRPN)	Bio X Cell	Cat# BE0088; RRID: AB_1107775
*InVivo*MAb Rat IgG2a isotype control (clone 2A3)	Bio X Cell	Cat# BE0089; RRID: AB_1107769
*InVivo*MAb Rat IgG2b isotype control (clone LTF-2)	Bio X Cell	Cat# BE0090; RRID: AB_1107780
*InVivo*MAb Syrian Hamster IgG isotype control (polyclonal)	Bio X Cell	Cat# BE0087; RRID: AB_1107782
Hamster anti-Mouse CD152/CTLA-4-PE (Clone UC10-4B9)	eBioscience	Cat# 12-1522-82; RRID: AB_465879
Rat anti-Mouse High Endothelial Venule-AF488 (Clone MECA-79)	eBioscience	Cat# 53-6036-82; RRID: AB_10804391
Rat anti-Mouse FoxP3-APC (Clone FJK-16s)	eBioscience	Cat# 17-5773-82; RRID: AB_469457
Rat anti-Mouse Ki67-eFluor 660 (Clone SoIA15)	eBioscience	Cat# 50-5698-82; RRID: AB_2574235
Rat anti-Mouse Ki67-AF488 (Clone SoIA15)	eBioscience	Cat# 53-5698-82; RRID: AB_2802330
Mouse anti-Mouse Tbet-PE (Clone 4B10)	eBioscience	Cat# 12-5825-82; RRID: AB_925761
Donkey anti-Rat IgG-AF647	Jackson ImmunoResearch	Cat# 712-605-153; RRID: AB_2340694
Goat anti-Rat IgM-AF488	Jackson ImmunoResearch	Cat# 112-547-020; RRID: AB_2632489
Goat anti-Rat IgM-Cy3	Jackson ImmunoResearch	Cat# 112-165-075; RRID: AB_2632489
Goat anti-Armenian Hamster IgG-AF488	Jackson ImmunoResearch	Cat# 127-545-160; RRID: AB_2338249
Mouse anti-Human CD32a/FcγRIIa-DyLight488 (Clone IV.3)	Smith et al.^55^	N/A
Mouse anti-Human CD32b/FcγRIIb-DyLight650 (Clone 2B6)	Smith et al.^55^	N/A
Mouse IgG2a anti-Mouse CTLA-4 (clone 9D9)	This paper	N/A
Mouse IgG1 D265A anti-Mouse CTLA-4 (clone 9D9)	This paper	N/A
Human IgG1 anti-Human CTLA-4 (clone 10D1, ipilimumab)	Knorr et al., 2024	N/A
Human IgG1 N297A anti-Human CTLA-4 (clone 10D1, ipilimumab)	Knorr et al.^35^	N/A
Human IgG1 GAALIE anti-Human CTLA-4 (clone 10D1, ipilimumab)	Knorr et al.^35^	N/A
Human IgG1 GAALIE isotype control (clone Z004)	This paper	N/A

**Chemicals, peptides, and recombinant proteins**		

MAXblock Blocking Medium	Active motif	Cat# 15252
OCT embedding matrix	CellPath	Cat# KMA-0100-00A
Protein G Sepharose 4 Fast Flow resin	Cytiva	Cat# 17061801
Fixable viability dye eFluor 506	Invitrogen	Cat# 65-0866-14
FoxP3/Transcription factor staining buffer set	Invitrogen	Cat# 00-5523-00
Streptavidin-DyLight550	Invitrogen	Cat# 84542
ACK lysing buffer	Lonza	Cat# 00-5523-00
Collagenase VIII	Sigma	Cat# C2139
DNaseI	Sigma	Cat# 11284932001

**Critical commercial assays**		

QuickChange II Site-Directed Mutagenesis Kit	Agilent	Cat# 200523
Cytofix/Cytoperm Fixation/Permeablization Kit	BD Biosciences	Cat# 554714
Cell Activation Cocktail (with Brefeldin A)	BioLegend	Cat# 423303
ExpiFectamine 293 Transfection Kit	Gibco	Cat# A14525
FoxP3/Transcription factor staining buffer set	Invitrogen	Cat# 00-5523-00

**Deposited data**		

scRNA-Seq	Asrir et al.^11^	GEO Series accession number - GEO: GSE154898

**Experimental models: Cell lines**		

B16F10 melanoma cells	ATCC	Cat# CRL-6475
Expi293F cells	Gibco	Cat# A14527
MCA-205 fibrosarcoma cells	Sigma	Cat# SCC173
MCA_prog_ (9609) fibrosarcoma cells	Asrir et al.^11^	Gift from R. Schreiber

**Experimental models: Organisms/strains**		

Mouse: C57BL/6	Charles River	Cat# 027
Mouse: C57BL/6 *Rag2*^−/−^	House Breeding	N/A
Mouse: C57BL/6 *Ctla4*^−/−^*CTLA4*^*tg*^	Peggs et al.^54^	N/A
Mouse: C57BL/6 FcRα^−/−^*Fcgr1*^−/−^*FCGR1*^*tg*^*FCGR2A*^*R131tg*^*FCGR2B*^*I232tg*^*FCGR3A*^*F158tg*^*FCGRIIIB*^*tg*^	Smith et al.^55^	N/A
Mouse: C57BL/6 *Ctla4*^−/−^*CTLA4*^*tg*^ FcRα^−/−^*Fcgr1*^−/−^*FCGR1*^*tg*^*FCGR2A*^*R131tg*^*FCGR2B*^*I232tg*^*FCGR3A*^*F158tg*^*FCGRIIIB*^*tg*^	Knorr et al.^35^	N/A

**Software and algorithms**		

DiVa v8.0.1	BD Biosciences	https://www.bdbiosciences.com
FlowJo v10.8.1	Tree Star	https://www.flowjo.com
GraphPad Prism 9.3.1	GraphPad	https://www.graphpad.com

**Other**		

Amino acid sequences of the variable regions of Mouse anti-Mouse CTLA-4 antibody (Clone 9D9)	Patent: US9868961B2	N/A
Amino acid sequences of the variable regions of Human anti-Human CTLA-4 antibody (Clone 10D1, ipilimumab)	NIH National Center for Advancing Translational Sciences Inxight Drugs	https://drugs.ncats.io/drug/6T8C155666

### Experimental model and study participant details

#### Animals

C57BL/6J mice were purchased from Charles River (Strain code #027). *Rag2*^−/−^ mice on a C57BL/6 background were obtained from European Mouse Mutant Archive (Strain code EM:00162). Humanized CTLA-4/FcγR mice (C57BL/6J background) were generated and bred at The Rockefeller University Comparative Bioscience Center. All animals were maintained in specific pathogen-free conditions. Animals were housed 2 to 5 per cage with unrestricted access to food and water. 6 to 12-week-old female mice were used except for experiments with humanized CTLA-4/FcγR mice for which both male and female were used. We have not analyzed the influence of biological sex on our findings. Thus, this is a limitation to the generalizability of the results. All mice were handled according to institutional guidelines under protocols approved by the French Ministry of Research and the FRBT (C2EA-01) animal care committee (Projects APAFIS#12297-2017112313506769v2 and APAFIS#23416-2019122019025727v3), and the Rockefeller University Institutional Animal Care and Use Committee (IACUC# 23018H).

#### Cell lines

Methylcholanthrene (MCA)-induced tumor-derived MCA-progressor (MCA_prog_) fibrosarcoma cells (9609) were provided by R. Schreiber (Washington University School of Medicine). B16F10 melanoma cells were obtained from ATCC (CRL-6475). MCA-205 cells were obtained from Sigma-Aldrich (SCC173). Cell lines were tested to be free of mycoplasma contamination but were not authenticated. MCA_prog_ cells were cultured in RPMI 1640 (SH30096.01, HyClone) with 10% Defined Fetal Bovine Serum (30070.03, HyClone). MCA-205 and B16F10 cells were culture in DMEM (Life Technologies) with 10% Fetal Bovine Serum (Life Technologies). All cells were cultured at 37°C under 5% CO_2_.

### Method details

#### Generation of humanized CTLA-4/FcγR mice

Humanized CTLA-4/FcγR mice were previously described.^35^ They were obtained by backcrossing humanized CTLA-4 mice into humanized FcγR mice. Humanized CTLA-4 mice were generated in the C57BL/6J background and characterized in previous studies.^54^ Briefly, transgenic mice expressing human CTLA-4 (expression of a chimeric construct containing upstream regulatory sequences required for mouse CTLA-4 expression and in which the extracellular coding domain of mouse CTLA-4 is replaced by that of human CTLA-4) were backcrossed into *Ctla4*^−/−^ mice that lack mouse CTLA-4. Humanized FcγR mice were generated in the C57BL/6 background and characterized in previous studies.^55^ Briefly, transgenic mice expressing human FCGR1A, FCGR2A^R131^, FCGR2B^I232^, FCGR3A^F158^ and FCGR3B (under the control of their human regulatory elements) were individually crossed together to create a mouse line expressing the full repertoire of human FcγRs. These human FcγR transgenic mice were then backcrossed into FcRα^−/−^ (deleted for *Fcgr2b*, *Fcgr3*, and *Fcgr4*) and *Fcgr1*^−/−^ mice that lack all mouse FcγRs.

#### Tumor transplantation

Tumor cells (0,5x10^6^) suspended in 100 μL DPBS were injected subcutaneously into the left flank of mice. Tumor growth was monitored by measuring the length and width of tumors with an electronic caliper. The tumor volume was estimated using the formula: (length x width^2^)/2, where length is the longest dimension. Tumor weight was determined at the end of the experiment and changes in tumor volume were determined using the formula: (volume day 12 - volume day 9)/volume day 9 x 100.

#### Antibody treatments

Anti-mouse CTLA-4 (clones 9D9 and 9H10), anti-human CTLA-4 (clone 10D1, ipilimumab), anti-mouse PD-1 (clone RMP1-14) and anti-mouse CD25 (clone PC-61.5.3) antibodies were administered intraperitoneally (ip.) at a dose of 200 μg per injection. In some experiments, mice were treated intratumorally (it.) with 50 μg of anti-mouse CTLA-4 (clone 9H10). Treatment schedule and endpoint are indicated in the figure legends. Fc-engineered antibodies were used similarly to their parental antibodies (same dose and treatment schedule). Mouse IgG2b (clone MPC-11), polyclonal Syrian hamster IgG, rat IgG2a (clone 2A3), rat IgG1 (clone HRPN), human IgG1 and human IgG1-GAALIE (clone Z004, human antibody targeting the envelope domain of Zika virus) antibodies were used as isotype controls. Antibodies were purchased from BioXCell except for Fc-engineered anti-mouse CTLA-4 antibodies (clone 9D9) containing mouse IgG2a or mouse IgG1 D265A Fc, human anti-CTLA-4 antibodies, and the human IgG1-GAALIE isotype control antibody that were produced in the laboratory of Jeffrey V. Ravetch at the Rockefeller University.

#### Production of recombinant antibodies and Fc-engineering

The sequences of the variable regions of the heavy and light chains of anti-mouse CTLA-4 antibody clone 9D9 and anti-human CTLA-4 antibody clone 10D1 (ipilimumab) were synthesized based on their published sequences, and subcloned into mammalian expression vectors containing mouse IgG or human IgG Fc backbones, as previously described.^57^^,^^58^ For the generation of Fc-domain variants of mouse IgG1 (D265A) and human IgG1 (N297A and GAALIE: G236A/A330L/I332E), site-directed mutagenesis using specific primers was performed according to the manufacturer’s instructions (QuickChange site-directed mutagenesis Kit II, Agilent Technologies). Mutated plasmid sequences were validated by direct sequencing. Antibodies were then generated by transient co-transfection of Expi293F cells with heavy-chain and light-chain expression plasmids. Expi293F cells were maintained in serum-free Expi293 Expression Medium and transfected using an ExpiFectamine 293 Transfection Kit (Thermo Fischer Scientific). Supernatants were collected 7 days after transfection, centrifuged, and filtered (0.22 μm). Antibodies were purified from clarified supernatants using Protein G Sepharose 4 Fast Flow (GE Healthcare), dialyzed in PBS, and sterile filtered (0.22 μm).

#### *In vivo* T cell depletion and cytokine neutralization

Anti-mouse CD4 (clone GK1.5) antibody was used for *in vivo* depletion of CD4^+^ T cells. Rat IgG2b antibody (clone LTF-2) was used as isotype control. Anti-mouse IFNγ antibody (clone XMG1.2) was used for *in vivo* neutralization of IFNγ. Rat IgG1 antibody (clone HRPN) was used as isotype control. Mice were treated ip. with 200 μg of antibodies on days 1, 2, 3, 6 and 9, and were analyzed at day 12. Depletion of CD4^+^ T cells was confirmed by flow cytometry analysis at the end of the experiment. All antibodies were purchased from BioXCell.

#### Preparation of single-cell suspensions from mouse tissues

Subcutaneous tumor and draining lymph node (inguinal) were removed using forceps, placed in digestion buffer (PBS with calcium and magnesium, 2% fetal bovine serum), and minced gently using scissors. Tissue fragments were digested in 1 mL (lymph node) or 3 mL (tumor) digestion buffer with collagenase type VIII (C2139, Sigma-Aldrich) and DNaseI (11284932001, Sigma-Aldrich) during 20 min (lymph node) or 30 min (tumor) at 37°C on a rocker. Concentrations were as follows: 1 mg/mL collagenase type VIII (lymph node and tumor), 10 μg/mL DNaseI (lymph node) or 100 μg/mL DNaseI (tumor). Digestion was halted by adding flow cytometry buffer (DPBS without calcium and magnesium, 2% fetal bovine serum, 2 mM EDTA) and cells were filtered through a 40 μm cell strainer (lymph node) or 70 μm cell strainer (tumor).

#### Flow cytometry

For cell surface staining, cells were resuspended in flow cytometry buffer containing 1:200 purified anti-CD16/32 antibody (clone 93, BioLegend) or Human TruStain FcX (BioLegend) plus antibodies to extracellular targets, and incubated for 30 min on ice in the dark. For intracellular staining (FOXP3, Tbet, Ki67, Granzyme B, CD25, and CTLA-4), cells were fixed and permeabilized using Foxp3 Transcription Factor Staining Buffer Set (00-5523-00, eBioscience). Following cell surface staining, cells were fixed with Fixation/Permeabilization solution for 20 min at room temperature in the dark and washed with Permeabilization buffer. Cells were then resuspended in Permeabilization buffer containing antibodies to intracellular targets and incubated for 45 min at room temperature in the dark.

The following anti-mouse antibodies were used: CD45-PerCP (clone 30-F11, BioLegend), CD11b-BV711 (clone M1/70, BioLegend), CD3e-PE-Cy7 (clone 17A2, BioLegend), CD8a-PB (clone 53–6.7, BioLegend), CD8a-FITC (clone 53–6.7, BioLegend), CD8a-APC-Cy7 (clone 53–6.7, BioLegend), CD4-AF488 (clone GK1.5, BioLegend), CD44-APC-Cy7 (clone IM7, BioLegend), PD-1-PE-Cy7 (clone 29F.1A12, BioLegend), ICOS-BV711 (clone C398.4A, BioLegend), CD39-PE-Cy7 (clone Duha59, BioLegend), FOXP3-APC (clone FJK-16s, eBioscience), Tbet-PE (clone 4B10, eBioscience), Ki67-AF488 (clone SolA15, eBioscience), Ki67-eFluor 660 (clone SolA15, eBioscience), Ki67-PB (clone 16A8, BioLegend), Granzyme B-PB (clone GB11, BioLegend), CTLA-4-PE (clone UC10-4B9, eBioscience), CTLA-4-BV421 (clone UC10-4B9, BioLegend), CD25-PE (clone PC61, BioLegend) IFNγ-AF647 (clone XMG1.2, BD Bioscience), CD31-PB (clone 390, BioLegend), High Endothelial Venule Marker-AF488 (clone MECA-79, eBioscience), PD-L1-Pe-Cy7 (clone 10F.9G2, BioLegend) and CD62P-AF647 (clone RB40.34, BD Biosciences). The following anti-human antibody was used: CTLA-4-PE (clone L3D10, BioLegend).

Fixable Viability Dye eFluor 506 (65-0866-14, eBioscience) was used to identify dead cells during analysis. Samples were acquired on BD LSRFortessa or Invitrogen Attune NxT flow cytometers and analyzed using FlowJo 10.8.1 (BD).

#### Analysis of FcγR expression on immune cells

Following viability staining, cells were incubated with antibodies to human FcγRs. Fluorochrome-conjugated anti-human antibodies were used as follows: CD64-PE (clone 10.1, BioLegend, used at 1:100 dilution), CD16-PE-Cy7 (clone 3G8, BioLegend, used at 1:100 dilution), CD32a-Dylight488 (clone IV.3, produced in the laboratory of Jeffrey V. Ravetch at the Rockefeller University, used at 10 μg/mL) and CD32b-Dylight650 (clone 2B6, produced in the laboratory of Jeffrey V. Ravetch at the Rockefeller University, used at 10 μg/mL). After 30min incubation at 4°C, cells were then incubated with other antibodies to cell surface targets and processed normally before acquisition on the flow cytometer.

#### *Ex vivo* analysis of IFNγ production by tumor-infiltrating T cells

Cells isolated from tumors were stimulated with PMA/Ionomycin cocktail containing Brefeldin A at 1:500 dilution (Biolegend, 423303) and incubated for 4 h at 37°C under 5% CO_2_. Cells were then stained for T cell surface markers as described above, and were fixed and permeabilized using Cytofix/Cytoperm kit (554714, BD Biosciences). Finally, cells were resuspended in Perm/Wash buffer containing anti-mouse IFNγ-AF647 (clone XMG1.2, BD Bioscience), and incubated for 45 min at room temperature in the dark. IFNγ expression was analyzed by flow cytometry.

#### Immunofluorescence staining

Resected tumors were fixed in paraformaldehyde 4% for 1 h at room temperature, and then incubated overnight in sucrose 30%. Samples were flash frozen in OCT embedding media using liquid nitrogen. Frozen samples were cut at 5–10 μm using a cryostat. Following brief warming to room temperature, tissues were rehydrated with PBS and incubated in MAXblock Blocking Medium (15252, Active Motif) for 1 h at room temperature. Tissues were then incubated with primary antibodies overnight at 4°C in 20% MAXblock Blocking Medium (DPBS). Slides were washed 3 times with DPBS, and tissues were incubated with secondary antibodies in 20% MAXblock Blocking Medium (DPBS) for 1 h at room temperature. For biotin-conjugated secondary antibodies, slides were washed 3 times with DPBS, and tissues were incubated with fluorescent Streptavidin conjugates in 20% MAXblock Blocking Medium (DPBS) for 1 h at room temperature. Slides were counterstained with DAPI and mounted in Mowiol. Images were taken using Zeiss wide-field microscope. Images of whole B16F10 tumor slides were taken using Akoya PhenoImager.

The following primary antibodies were used: rat anti-mouse CD31 (clone MEC 13.3, BD Biosciences), rat anti-human/mouse High Endothelial Venule (clone MECA-79, hybridoma supernatant), biotin-conjugated rat anti-human/mouse High Endothelial Venule (clone MECA-79, BioLegend), and Armenian hamster anti-mouse CD3e (clone 145-2C11, BD Biosciences).

The following secondary antibodies and fluorescent streptavidin conjugate were used: goat anti-rat IgM-AF488 (112-547-020, Jackson ImmunoResearch), goat anti-rat IgM-Cy3 (112-165-075, Jackson ImmunoResearch), donkey anti-rat IgG-AF647 (712-605-153, Jackson ImmunoResearch), goat anti-Armenian hamster IgG-AF488 (127-545-160, Jackson ImmunoResearch), and streptavidin-Dylight550 (84542, Invitrogen).

### Quantification and statistical analysis

For tumor growth experiments, statistical significance was determined using two-way ANOVA tests. Analyses of overall survival were performed using log rank tests. Correlation analyses were performed using Pearson correlation tests. For other experiments, unpaired two-tailed Student’s t-tests were used to calculate statistical differences between two groups, and one-way ANOVA tests were used to calculate statistical differences between multiple groups. *p* values were defined as follows: ∗*p* < 0.05; ∗∗*p* < 0.01; ∗∗∗*p* < 0.001; ∗∗∗∗*p* < 0.0001; ns, not significant. All quantitative data were obtained from at least two independent experiments. Data in graphs are presented as mean ± SEM. Statistical details of experiments can be found in the figure legends, including the statistical tests used, number of pooled experiments, exact value of n and what n represents. Experimental data were analyzed and visualized using GraphPad Prism software (version 9.3.1; San Diego, CA, USA).

## Supplemental information

Document S1. Figures S1–S12

Document S2. Article plus supplemental information

## References

[1] Ribas A., Wolchok J.D. (2018). Cancer immunotherapy using checkpoint blockade. Science.

[2] Sharma P., Siddiqui B.A., Anandhan S., Yadav S.S., Subudhi S.K., Gao J., Goswami S., Allison J.P. (2021). The Next Decade of Immune Checkpoint Therapy. Cancer Discov..

[3] Gajewski T.F. (2015). The Next Hurdle in Cancer Immunotherapy: Overcoming the Non-T-Cell-Inflamed Tumor Microenvironment. Semin. Oncol..

[4] Galon J., Bruni D. (2019). Approaches to treat immune hot, altered and cold tumours with combination immunotherapies. Nat. Rev. Drug Discov..

[5] Tumeh P.C., Harview C.L., Yearley J.H., Shintaku I.P., Taylor E.J.M., Robert L., Chmielowski B., Spasic M., Henry G., Ciobanu V. (2014). PD-1 blockade induces responses by inhibiting adaptive immune resistance. Nature.

[6] Grasso C.S., Tsoi J., Onyshchenko M., Abril-Rodriguez G., Ross-Macdonald P., Wind-Rotolo M., Champhekar A., Medina E., Torrejon D.Y., Shin D.S. (2020). Conserved Interferon-γ Signaling Drives Clinical Response to Immune Checkpoint Blockade Therapy in Melanoma. Cancer Cell.

[7] Newell F., Pires da Silva I., Johansson P.A., Menzies A.M., Wilmott J.S., Addala V., Carlino M.S., Rizos H., Nones K., Edwards J.J. (2022). Multiomic profiling of checkpoint inhibitor-treated melanoma: Identifying predictors of response and resistance, and markers of biological discordance. Cancer Cell.

[8] Girard J.-P., Moussion C., Förster R. (2012). HEVs, lymphatics and homeostatic immune cell trafficking in lymph nodes. Nat. Rev. Immunol..

[9] Martinet L., Garrido I., Filleron T., Le Guellec S., Bellard E., Fournie J.-J., Rochaix P., Girard J.-P. (2011). Human solid tumors contain high endothelial venules: association with T- and B-lymphocyte infiltration and favorable prognosis in breast cancer. Cancer Res..

[10] Blanchard L., Girard J.P. (2021). High endothelial venules (HEVs) in immunity, inflammation and cancer. Angiogenesis.

[11] Asrir A., Tardiveau C., Coudert J., Laffont R., Blanchard L., Bellard E., Veerman K., Bettini S., Lafouresse F., Vina E. (2022). Tumor-associated high endothelial venules mediate lymphocyte entry into tumors and predict response to PD-1 plus CTLA-4 combination immunotherapy. Cancer Cell.

[12] Hua Y., Vella G., Rambow F., Allen E., Antoranz Martinez A., Duhamel M., Takeda A., Jalkanen S., Junius S., Smeets A. (2022). Cancer immunotherapies transition endothelial cells into HEVs that generate TCF1+ T lymphocyte niches through a feed-forward loop. Cancer Cell.

[13] Sautès-Fridman C., Petitprez F., Calderaro J., Fridman W.H. (2019). Tertiary lymphoid structures in the era of cancer immunotherapy. Nat. Rev. Cancer.

[14] Ramachandran M., Vaccaro A., van de Walle T., Georganaki M., Lugano R., Vemuri K., Kourougkiaouri D., Vazaios K., Hedlund M., Tsaridou G. (2023). Tailoring vascular phenotype through AAV therapy promotes anti-tumor immunity in glioma. Cancer Cell.

[15] Moussion C., Girard J.-P. (2011). Dendritic cells control lymphocyte entry to lymph nodes through high endothelial venules. Nature.

[16] Tang H., Wang Y., Chlewicki L.K., Zhang Y., Guo J., Liang W., Wang J., Wang X., Fu Y.X. (2016). Facilitating T Cell Infiltration in Tumor Microenvironment Overcomes Resistance to PD-L1 Blockade. Cancer Cell.

[17] Johansson-Percival A., He B., Li Z.J., Kjellén A., Russell K., Li J., Larma I., Ganss R. (2017). De novo induction of intratumoral lymphoid structures and vessel normalization enhances immunotherapy in resistant tumors. Nat. Immunol..

[18] Allen E., Jabouille A., Rivera L.B., Lodewijckx I., Missiaen R., Steri V., Feyen K., Tawney J., Hanahan D., Michael I.P., Bergers G. (2017). Combined antiangiogenic and anti-PD-L1 therapy stimulates tumor immunity through HEV formation. Sci. Transl. Med..

[19] Drayton D.L., Liao S., Mounzer R.H., Ruddle N.H. (2006).

[20] Wei S.C., Duffy C.R., Allison J.P. (2018). Fundamental Mechanisms of Immune Checkpoint Blockade Therapy. Cancer Discov..

[21] Kvistborg P., Philips D., Kelderman S., Hageman L., Ottensmeier C., Joseph-Pietras D., Welters M.J.P., Van Der Burg S., Kapiteijn E., Michielin O. (2014). Anti-CTLA-4 therapy broadens the melanoma-reactive CD8+ T cell response. Sci. Transl. Med..

[22] Wei S.C., Levine J.H., Cogdill A.P., Zhao Y., Anang N.A.A.S., Andrews M.C., Sharma P., Wang J., Wargo J.A., Pe’er D., Allison J.P. (2017). Distinct Cellular Mechanisms Underlie Anti-CTLA-4 and Anti-PD-1 Checkpoint Blockade. Cell.

[23] Wei S.C., Sharma R., Anang N.A.A.S., Levine J.H., Zhao Y., Mancuso J.J., Setty M., Sharma P., Wang J., Pe’er D., Allison J.P. (2019). Negative Co-stimulation Constrains T Cell Differentiation by Imposing Boundaries on Possible Cell States. Immunity.

[24] Liakou C.I., Kamat A., Tang D.N., Chen H., Sun J., Troncoso P., Logothetis C., Sharma P. (2008). CTLA-4 blockade increases IFNgamma-producing CD4+ICOShi cells to shift the ratio of effector to regulatory T cells in cancer patients. Proc. Natl. Acad. Sci. USA.

[25] Ng Tang D., Shen Y., Sun J., Wen S., Wolchok J.D., Yuan J., Allison J.P., Sharma P. (2013). Increased frequency of ICOS+ CD4 T cells as a pharmacodynamic biomarker for anti-CTLA-4 therapy. Cancer Immunol. Res..

[26] Fu T., He Q., Sharma P. (2011). The ICOS/ICOSL pathway is required for optimal antitumor responses mediated by anti-CTLA-4 therapy. Cancer Res..

[27] Simpson T.R., Li F., Montalvo-Ortiz W., Sepulveda M.A., Bergerhoff K., Arce F., Roddie C., Henry J.Y., Yagita H., Wolchok J.D. (2013). Fc-dependent depletion of tumor-infiltrating regulatory T cells co-defines the efficacy of anti-CTLA-4 therapy against melanoma. J. Exp. Med..

[28] Bulliard Y., Jolicoeur R., Windman M., Rue S.M., Ettenberg S., Knee D.A., Wilson N.S., Dranoff G., Brogdon J.L. (2013). Activating Fc γ receptors contribute to the antitumor activities of immunoregulatory receptor-targeting antibodies. J. Exp. Med..

[29] Selby M.J., Engelhardt J.J., Quigley M., Henning K.A., Chen T., Srinivasan M., Korman A.J. (2013). Anti-CTLA-4 antibodies of IgG2a isotype enhance antitumor activity through reduction of intratumoral regulatory T cells. Cancer Immunol. Res..

[30] Ingram J.R., Blomberg O.S., Rashidian M., Ali L., Garforth S., Fedorov E., Fedorov A.A., Bonanno J.B., Le Gall C., Crowley S. (2018). Anti-CTLA-4 therapy requires an Fc domain for efficacy. Proc. Natl. Acad. Sci. USA.

[31] Arce Vargas F., Furness A.J.S., Litchfield K., Joshi K., Rosenthal R., Ghorani E., Solomon I., Lesko M.H., Ruef N., Roddie C. (2018). Fc Effector Function Contributes to the Activity of Human Anti-CTLA-4 Antibodies. Cancer Cell.

[32] Lax B.M., Palmeri J.R., Lutz E.A., Sheen A., Stinson J.A., Duhamel L., Santollani L., Kennedy A., Rothschilds A.M., Spranger S. (2023). Both intratumoral regulatory T cell depletion and CTLA-4 antagonism are required for maximum efficacy of anti-CTLA-4 antibodies. Proc. Natl. Acad. Sci. USA.

[33] Sharma A., Subudhi S.K., Blando J., Scutti J., Vence L., Wargo J., Allison J.P., Ribas A., Sharma P. (2019). Anti-CTLA-4 Immunotherapy Does Not Deplete FOXP3 + Regulatory T Cells (Tregs) in Human Cancers. Clin. Cancer Res..

[34] Quezada S.A., Peggs K.S. (2019). Lost in Translation: Deciphering the Mechanism of Action of Anti-human CTLA-4. Clin. Cancer Res..

[35] Knorr D.A., Blanchard L., Leidner R.S., Jensen S.M., Meng R., Jones A., Ballesteros-Merino C., Bell R.B., Baez M., Marino A. (2024). FcγRIIB Is an Immune Checkpoint Limiting the Activity of Treg-Targeting Antibodies in the Tumor Microenvironment. Cancer Immunol. Res..

[36] Waight J.D., Chand D., Dietrich S., Gombos R., Horn T., Gonzalez A.M., Manrique M., Swiech L., Morin B., Brittsan C. (2018). Selective FcγR Co-engagement on APCs Modulates the Activity of Therapeutic Antibodies Targeting T Cell Antigens. Cancer Cell.

[37] Yofe I., Landsberger T., Yalin A., Solomon I., Costoya C., Demane D.F., Shah M., David E., Borenstein C., Barboy O. (2022). Anti-CTLA-4 antibodies drive myeloid activation and reprogram the tumor microenvironment through FcγR engagement and type I interferon signaling. Nat. Cancer.

[38] Sharma N., Fan X., Atolagbe O.T., Ge Z., Dao K.N., Sharma P., Allison J.P. (2024). ICOS costimulation in combination with CTLA-4 blockade remodels tumor-associated macrophages toward an antitumor phenotype. J. Exp. Med..

[39] Blanchard L., Vina E., Asrir A., Tardiveau C., Coudert J., Laffont R., Tarroux D., Bettini S., Veerman K., Lafouresse F. (2022). Flow cytometry analysis of endothelial cells and subsets of exhausted CD8+ T cells in murine tumor models. STAR Protoc..

[40] Nimmerjahn F., Ravetch J.V. (2005). Divergent immunoglobulin g subclass activity through selective Fc receptor binding. Science.

[41] Nimmerjahn F., Bruhns P., Horiuchi K., Ravetch J.V. (2005). FcgammaRIV: a novel FcR with distinct IgG subclass specificity. Immunity.

[42] Zappasodi R., Serganova I., Cohen I.J., Maeda M., Shindo M., Senbabaoglu Y., Watson M.J., Leftin A., Maniyar R., Verma S. (2021). CTLA-4 blockade drives loss of Treg stability in glycolysis-low tumours. Nature.

[43] Koch M.A., Tucker-Heard G., Perdue N.R., Killebrew J.R., Urdahl K.B., Campbell D.J. (2009). The transcription factor T-bet controls regulatory T cell homeostasis and function during type 1 inflammation. Nat. Immunol..

[44] Geels S.N., Moshensky A., Sousa R.S., Murat C., Bustos M.A., Walker B.L., Singh R., Harbour S.N., Gutierrez G., Hwang M. (2024). Interruption of the intratumor CD8+ T cell:Treg crosstalk improves the efficacy of PD-1 immunotherapy. Cancer Cell.

[45] Tay C., Tanaka A., Sakaguchi S. (2023). Tumor-infiltrating regulatory T cells as targets of cancer immunotherapy. Cancer Cell.

[46] Chen H., Fu T., Suh W.K., Tsavachidou D., Wen S., Gao J., Ng Tang D., He Q., Sun J., Sharma P. (2014). CD4 T cells require ICOS-mediated PI3K signaling to increase T-Bet expression in the setting of anti-CTLA-4 therapy. Cancer Immunol. Res..

[47] Johansson A., Hamzah J., Payne C.J., Ganss R. (2012). Tumor-targeted TNFα stabilizes tumor vessels and enhances active immunotherapy. Proc. Natl. Acad. Sci. USA.

[48] Kammertoens T., Friese C., Arina A., Idel C., Briesemeister D., Rothe M., Ivanov A., Szymborska A., Patone G., Kunz S. (2017). Tumour ischaemia by interferon-γ resembles physiological blood vessel regression. Nature.

[49] Duhen R., Fesneau O., Samson K.A., Frye A.K., Beymer M., Rajamanickam V., Ross D., Tran E., Bernard B., Weinberg A.D., Duhen T. (2022). PD-1 and ICOS coexpression identifies tumor-reactive CD4+ T cells in human solid tumors. J. Clin. Investig..

[50] Wang K., Coutifaris P., Brocks D., Wang G., Azar T., Solis S., Nandi A., Anderson S., Han N., Manne S. (2024). Combination anti-PD-1 and anti-CTLA-4 therapy generates waves of clonal responses that include progenitor-exhausted CD8+ T cells. Cancer Cell.

[51] Tan C.L., Kuchroo J.R., Sage P.T., Liang D., Francisco L.M., Buck J., Thaker Y.R., Zhang Q., McArdel S.L., Juneja V.R. (2021). PD-1 restraint of regulatory T cell suppressive activity is critical for immune tolerance. J. Exp. Med..

[52] Perry J.A., Shallberg L., Clark J.T., Gullicksrud J.A., DeLong J.H., Douglas B.B., Hart A.P., Lanzar Z., O’Dea K., Konradt C. (2022). PD-L1-PD-1 interactions limit effector regulatory T cell populations at homeostasis and during infection. Nat. Immunol..

[53] Bournazos S., DiLillo D.J., Ravetch J.V. (2014). humanized mice to study FcγR function. Curr. Top. Microbiol. Immunol..

[54] Peggs K.S., Quezada S.A., Chambers C.A., Korman A.J., Allison J.P. (2009). Blockade of CTLA-4 on both effector and regulatory T cell compartments contributes to the antitumor activity of anti-CTLA-4 antibodies. J. Exp. Med..

[55] Smith P., DiLillo D.J., Bournazos S., Li F., Ravetch J.V. (2012). Mouse model recapitulating human Fcγ receptor structural and functional diversity. Proc. Natl. Acad. Sci. USA.

[56] Shields R.L., Namenuk A.K., Hong K., Meng Y.G., Rae J., Briggs J., Xie D., Lai J., Stadlen A., Li B. (2001). High resolution mapping of the binding site on human IgG1 for Fc gamma RI, Fc gamma RII, Fc gamma RIII, and FcRn and design of IgG1 variants with improved binding to the Fc gamma R. J. Biol. Chem..

[57] Weitzenfeld P., Bournazos S., Ravetch J.V. (2019). Antibodies targeting sialyl Lewis A mediate tumor clearance through distinct effector pathways. J. Clin. Investig..

[58] Bournazos S., Corti D., Virgin H.W., Ravetch J.V. (2020). Fc-optimized antibodies elicit CD8 immunity to viral respiratory infection. Nature.

[59] Curran M.A., Montalvo W., Yagita H., Allison J.P. (2010). PD-1 and CTLA-4 combination blockade expands infiltrating T cells and reduces regulatory T and myeloid cells within B16 melanoma tumors. Proc. Natl. Acad. Sci. USA.

[60] Peske J.D., Thompson E.D., Gemta L., Baylis R.A., Fu Y.X., Engelhard V.H. (2015). Effector lymphocyte-induced lymph node-like vasculature enables naive T-cell entry into tumours and enhanced anti-tumour immunity. Nat. Commun..

[61] Tian L., Goldstein A., Wang H., Ching Lo H., Sun Kim I., Welte T., Sheng K., Dobrolecki L.E., Zhang X., Putluri N. (2017). Mutual regulation of tumour vessel normalization and immunostimulatory reprogramming. Nature.

[62] Zheng X., Fang Z., Liu X., Deng S., Zhou P., Wang X., Zhang C., Yin R., Hu H., Chen X. (2018). Increased vessel perfusion predicts the efficacy of immune checkpoint blockade. J. Clin. Investig..

[63] Ayers M., Lunceford J., Nebozhyn M., Murphy E., Loboda A., Kaufman D.R., Albright A., Cheng J.D., Kang S.P., Shankaran V. (2017). IFN-γ–related mRNA profile predicts clinical response to PD-1 blockade. J. Clin. Investig..

[64] Benci J.L., Xu B., Qiu Y., Wu T.J., Dada H., Twyman-Saint Victor C., Cucolo L., Lee D.S.M., Pauken K.E., Huang A.C. (2016). Tumor Interferon Signaling Regulates a Multigenic Resistance Program to Immune Checkpoint Blockade. Cell.

[65] Qiu J., Xu B., Ye D., Ren D., Wang S., Benci J.L., Xu Y., Ishwaran H., Beltra J.C., Wherry E.J. (2023). Cancer cells resistant to immune checkpoint blockade acquire interferon-associated epigenetic memory to sustain T cell dysfunction. Nat. Cancer.

[66] Rudqvist N.P., Avagyan M., Chand D. (2023). Next-generation CTLA-4 targeting molecules and combination therapy: promising strategies for improving cancer immunotherapy. OncoImmunology.

[67] Wei S.C., Sharma R., Anang N.A.A.S., Levine J.H., Zhao Y., Mancuso J.J., Setty M., Sharma P., Wang J., Pe’er D., Allison J.P. (2019). Negative Co-stimulation Constrains T Cell Differentiation by Imposing Boundaries on Possible Cell States. Immunity.

[68] Thibaut R., Bost P., Milo I., Cazaux M., Lemaître F., Garcia Z., Amit I., Breart B., Cornuot C., Schwikowski B., Bousso P. (2020). Bystander IFN-γ activity promotes widespread and sustained cytokine signaling altering the tumor microenvironment. Nat. Cancer.

[69] Hoekstra M.E., Bornes L., Dijkgraaf F.E., Philips D., Pardieck I.N., Toebes M., Thommen D.S., van Rheenen J., Schumacher T.N.M. (2020). Long-distance modulation of bystander tumor cells by CD8+ T cell-secreted IFNγ. Nat. Cancer.

[70] Li C.W., Lim S.O., Xia W., Lee H.H., Chan L.C., Kuo C.W., Khoo K.H., Chang S.S., Cha J.H., Kim T. (2016). Glycosylation and stabilization of programmed death ligand-1 suppresses T-cell activity. Nat. Commun..

[71] Li C.W., Lim S.O., Chung E.M., Kim Y.S., Park A.H., Yao J., Cha J.H., Xia W., Chan L.C., Kim T. (2018). Eradication of Triple-Negative Breast Cancer Cells by Targeting Glycosylated PD-L1. Cancer Cell.

[72] Yeh J.C., Hiraoka N., Petryniak B., Nakayama J., Ellies L.G., Rabuka D., Hindsgaul O., Marth J.D., Lowe J.B., Fukuda M. (2001). Novel sulfated lymphocyte homing receptors and their control by a core1 extension β1,3-N-acetylglucosaminyltransferase. Cell.

[73] Bullock A.J., Schlechter B.L., Fakih M.G., Tsimberidou A.M., Grossman J.E., Gordon M.S., Wilky B.A., Pimentel A., Mahadevan D., Balmanoukian A.S. (2024). Botensilimab plus balstilimab in relapsed/refractory microsatellite stable metastatic colorectal cancer: a phase 1 trial. Nat. Med..

[74] Wilky B.A., Schwartz G.K., Gordon M.S., El-Khoueiry A.B., Bullock A.J., Henick B., Agulnik M., Singh A., Mahadevan D., Stebbing J. (2025). Botensilimab (Fc-enhanced anti-cytotoxic lymphocyte-association protein-4 antibody) Plus Balstilimab (anti-PD-1 antibody) in Patients With Relapsed/Refractory Metastatic Sarcomas. J. Clin. Oncol..

[75] He B., Johansson-Percival A., Backhouse J., Li J., Lee G.Y.F., Hamzah J., Ganss R. (2020). Remodeling of Metastatic Vasculature Reduces Lung Colonization and Sensitizes Overt Metastases to Immunotherapy. Cell Rep..

