# Supplementary material for: Fc-optimized anti-CTLA-4 antibodies increase tumor-associated high endothelial venules and sensitize refractory tumors to PD-1 blockade
Source: Cell Rep Med. 2025 Jun 3;6(6):102141. doi: 10.1016/j.xcrm.2025.102141 (PMC12208319; doi:10.1016/j.xcrm.2025.102141)
Supplement: Document S1. Figures S1–S12 [file mmc1.pdf]

Cell Reports Medicine, Volume *6*

Supplemental information

Fc-optimized anti-CTLA-4 antibodies increase tumor-associated high endothelial venules and sensitize refractory tumors to PD-1 blockade

Lucas Blanchard, Estefania Vina, Jerko Ljubetic, Cécile Meneur, Dorian Tarroux, Maria Baez, Alessandra Marino, Nathalie Ortega, David A. Knorr, Jeffrey V. Ravetch, and Jean-Philippe Girard

Figure S1

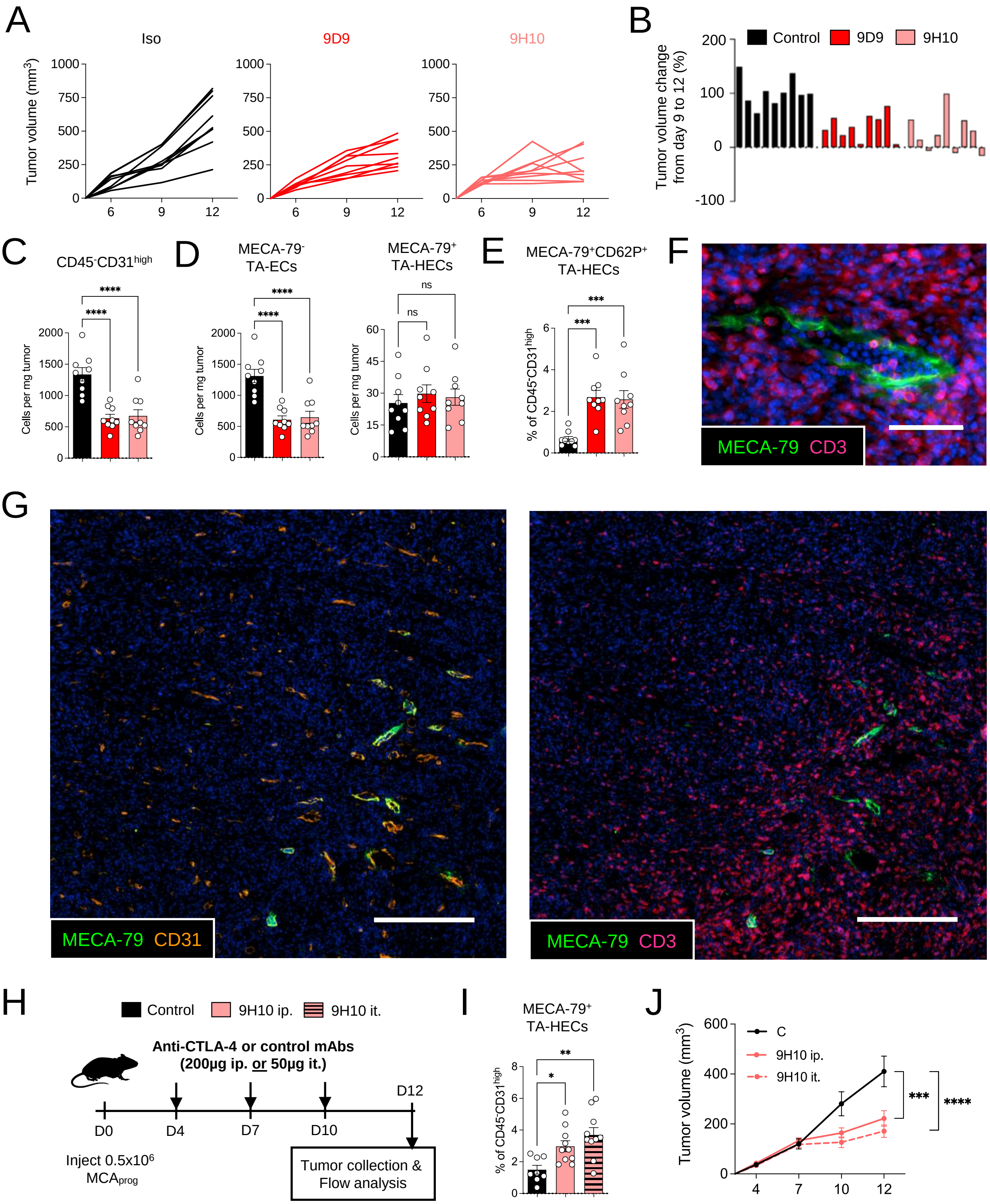

A
Iso
9D9
9H10
Tumor volume (mm3)
B
Control
9D9
9H10
Tumor volume change from day 9 to 12 (%)
C
CD45-CD31high
Cells per mg tumor
D
MECA-79- TA-ECs
MECA-79+ TA-HECs
E
MECA-79+CD62P+ TA-HECs
% of CD45-CD31high
F
MECA-79 CD3
G
MECA-79 CD31
MECA-79 CD3
H
Control
9H10 ip.
9H10 it.
Anti-CTLA-4 or control mAbs (200µg ip. or 50µg it.)
D0
D4
D7
D10
D12
Inject 0.5x10⁶ MCAprog
Tumor collection & Flow analysis
I
MECA-79+ TA-HECs
J
C
9H10 ip.
9H10 it.
Tumor volume (mm3)

Figure S1. Anti-CTLA-4 antibodies modulate TA-HEVs, Related to Figure 1.
(A and B) Individual tumor growth and tumor volume changes in individual mice. Each bar represents an individual mouse. Data were obtained from two independent experiments (total: 9 mice per group). Data were obtained from two independent experiments (total: 9 mice per group).
(C-E) Numbers of $CD45^{-}CD31^{high}$ endothelial cells, TA-ECs and TA-HECs, and frequency of MECA-$79^{+}CD62P^{+}$ TA-HECs. Each symbol represents an individual mouse. Data were obtained from two independent experiments (total: 9 mice per group).
(F and G) Immunofluorescence of MCAprog tumor (day 12) following treatment with 9D9 mouse anti-CTLA-4 antibody. Selected markers are presented. Scale bars: 50 µm in (F), and 200 µm in (G).
(H) Treatment schedule. ip., intraperitoneal. it., intratumoral. Isotype control antibodies were used in the control group.
(I and J) Frequency of TA-HECs and mean tumor growth. Each symbol represents an individual mouse. Data were obtained from two independent experiments (total: Control = 8 mice, 9H10 ip. = 10 mice, 9H10 it. = 10 mice).
Data are shown as mean ± SEM. All p values were determined by one-way ANOVA with Tukey's multiple comparison test, except for panel (J) in which p values were determined by two-way ANOVA with Dunnet's multiple comparison test.

Figure S2

A

MOUSE Fc-FcγRs	FcγRIIb (inhibitory)	FcγRIII (activating)	FcγRIV (activating)	A/I ratio
Mouse IgG1	++	+	-	0,1
Mouse IgG2b	++	+	+++	7
Mouse IgG1 D265A	-	-	-	ND
Hamster IgG2b	ND	++	++++	ND

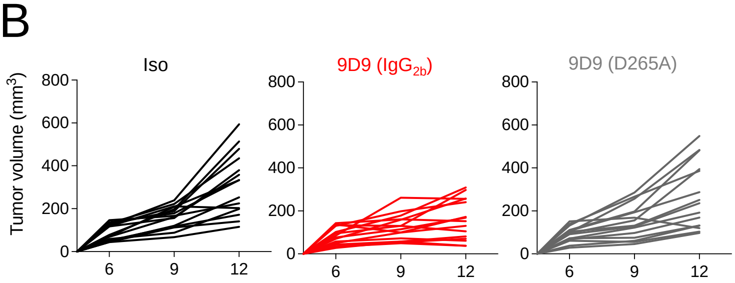

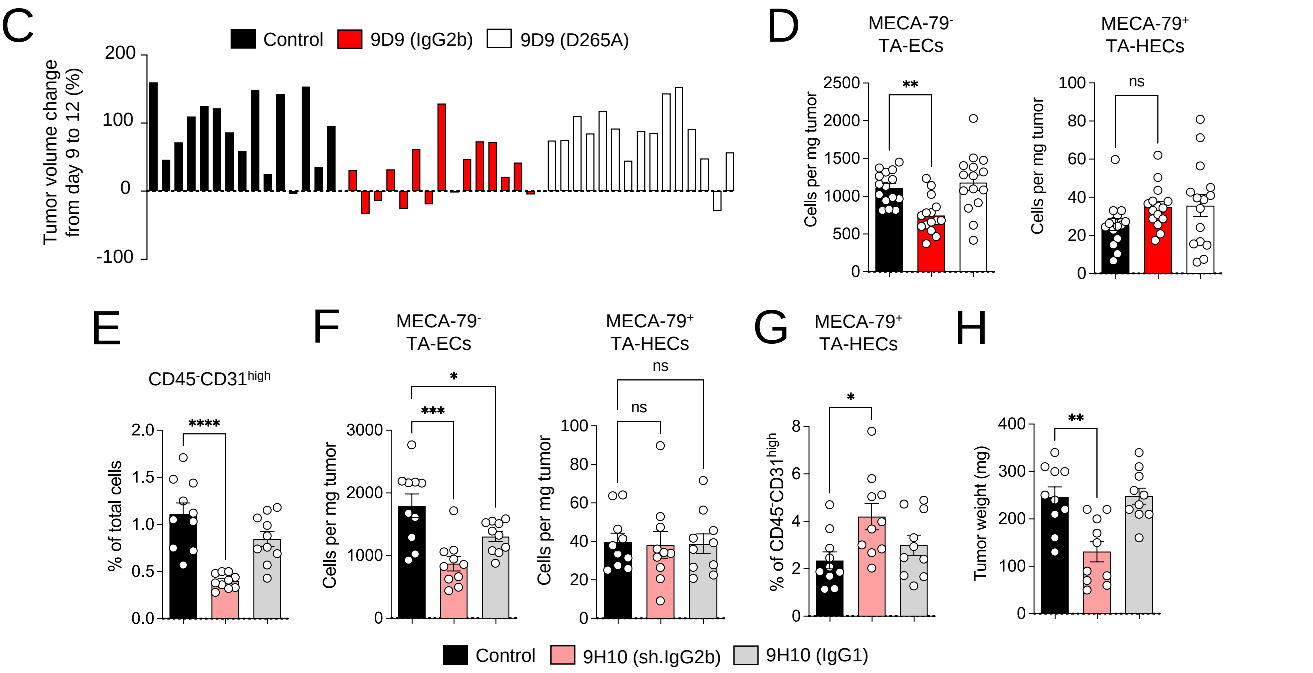

Figure S2. Anti-CTLA-4 antibodies modulate tumor endothelial cells and TA-HEVs through Fc-dependent mechanisms, Related to Figure 1.

(A) Table presenting the binding profiles to mouse FcγRs of the various IgGs used in the study. Relative binding affinities are proposed based on affinity constants previously assessed by surface plasmon resonance or other binding assays [S1-S3]. The relative Fc effector function of mouse IgGs is presented as the ratio of activating (FcγRIII for mouse IgG1; FcγRIV for mouse IgG2b) to inhibitory FcγR (FcγRIIb) binding.

(B and C) Individual tumor growth and tumor volume changes in individual mice. Each bar represents an individual mouse. Data were obtained from three independent experiments (total: 15 mice per group).

(D) Numbers of TA-ECs and TA-HECs. Each symbol represents an individual mouse. Data were obtained from three independent experiments (total: 15 mice per group).

(E-H) Frequency of $CD45^{-}CD31^{high}$ endothelial cells, numbers of TA-ECs and TA-HECs, frequency of TA-HECs and tumor weights in individual mice. Each symbol represents an individual mouse. Data were obtained from two independent experiments (total: Control = 8 mice, 9H10 ip. = 10 mice, 9H10 it. = 10 mice).

Data are shown as mean ± SEM. All p values were determined by one-way ANOVA with Tukey's multiple comparison test.

Figure S3

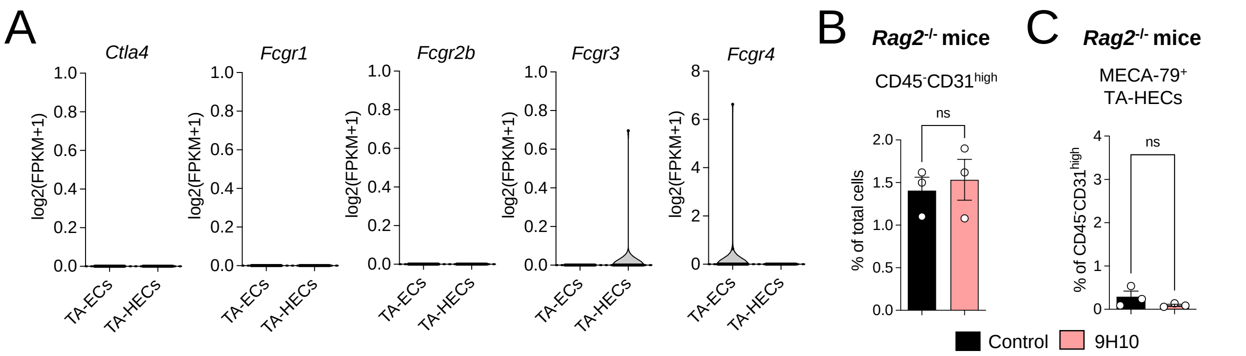

Figure S3. Anti-CTLA-4 antibodies require lymphocytes to modulate tumor endothelial cells and TA-HEVs, Related to Figure 2.

(A) Violin plots showing the expression distribution of selected genes in tumor-associated $CD45^{-}CD31^{high}MECA\text{-}79^{+}$ HEV endothelial cells (TA-HECs) and $CD45^{-}CD31^{high}MECA\text{-}79^{-}$ endothelial cells (TA-ECs). Gene expression values were obtained from single cell RNA-seq analyses of mRNA expression and calculated as fragments per kilobase of transcript per million (FKPM). y axis indicates log2 (FPKM+1) expression levels. Presented data were obtained from Asrir et al. [S4].

(B and C) Frequencies of $CD45^{-}CD31^{high}$ endothelial cells and TA-HECs. This experiment was performed in *Rag2*$^{-/-}$ mice. Each symbol represents an individual mouse. Data were obtained from one experiment (total: 3 mice per group).

Data are shown as mean ± SEM. All p values were determined by unpaired two-tailed Student’s t test.

Figure S4

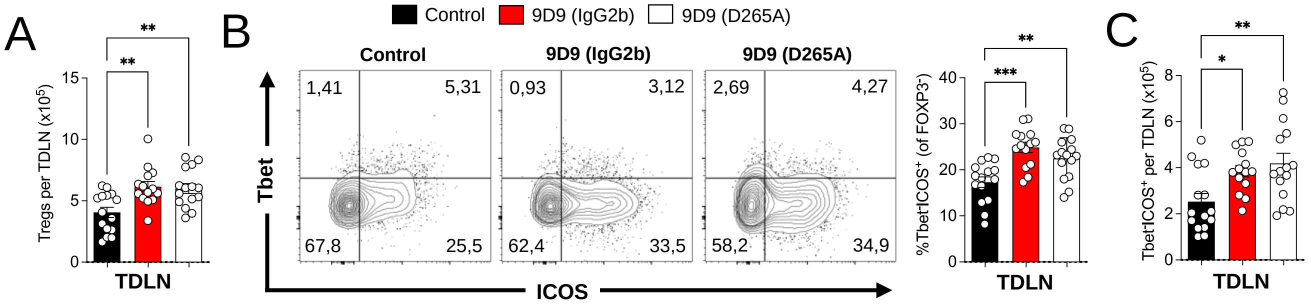

Figure S4. Anti-CTLA-4 antibodies modulate CD4⁺ T cells in tumor-draining lymph node independently of Fc effector function, Related to Figure 2.

(A) Number of FOXP3⁺ Tregs in tumor-draining lymph node (TDLN). Each symbol represents an individual mouse. Data were obtained from three independent experiments (total: Control = 15 mice, 9D9 = 14 mice, Fc-null 9D9= 15 mice).

(B and C) Frequency and number of Tbet⁻ICOS⁺ CD4⁺ T cells in TDLN following indicated treatments. Representative dot plots are shown (B). Each symbol represents an individual mouse. Data were obtained from three independent experiments (total: Control = 15 mice, 9D9 = 14 mice, Fc-null 9D9= 15 mice).

Data are shown as mean ± SEM. All p values were determined by one-way ANOVA with Tukey's multiple comparison.

Figure S5

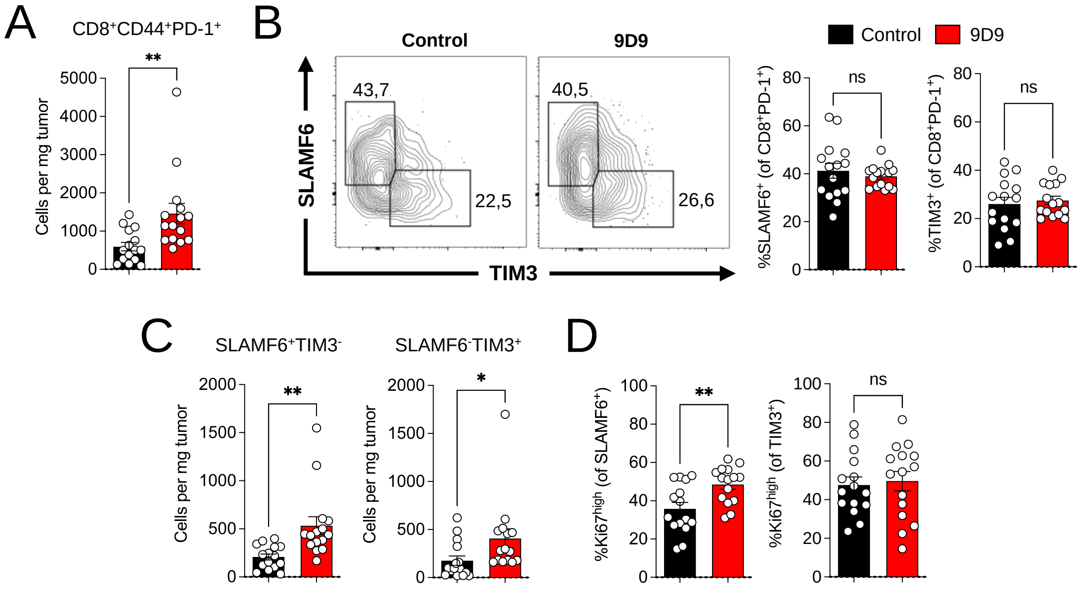

Figure S5. Anti-CTLA-4 antibodies modulate tumor-infiltrating CD8⁺ T cells, Related to Figure 2.
(A) Number of tumor-infiltrating exhausted CD44⁺PD-1⁺ CD8⁺ T cells. Each symbol represents an individual mouse. Data were obtained from three independent experiments (total: 15 mice per group).
(B-D) Frequencies and numbers of stem-like/progenitor exhausted (SLAMF6⁺TIM3⁻) and effector/terminally exhausted (SLAMF6⁻TIM3⁺) CD8⁺ T cells in tumors, and frequencies of Ki67^{high} cells in these two subsets, following indicated treatments. Representative dot plots are shown (B). Each symbol represents an individual mouse. Data were obtained from three independent experiments (total: Control = 15 mice per group).
Data are shown as mean ± SEM. All p values were determined by unpaired two-tailed Student’s t test.

Figure S6

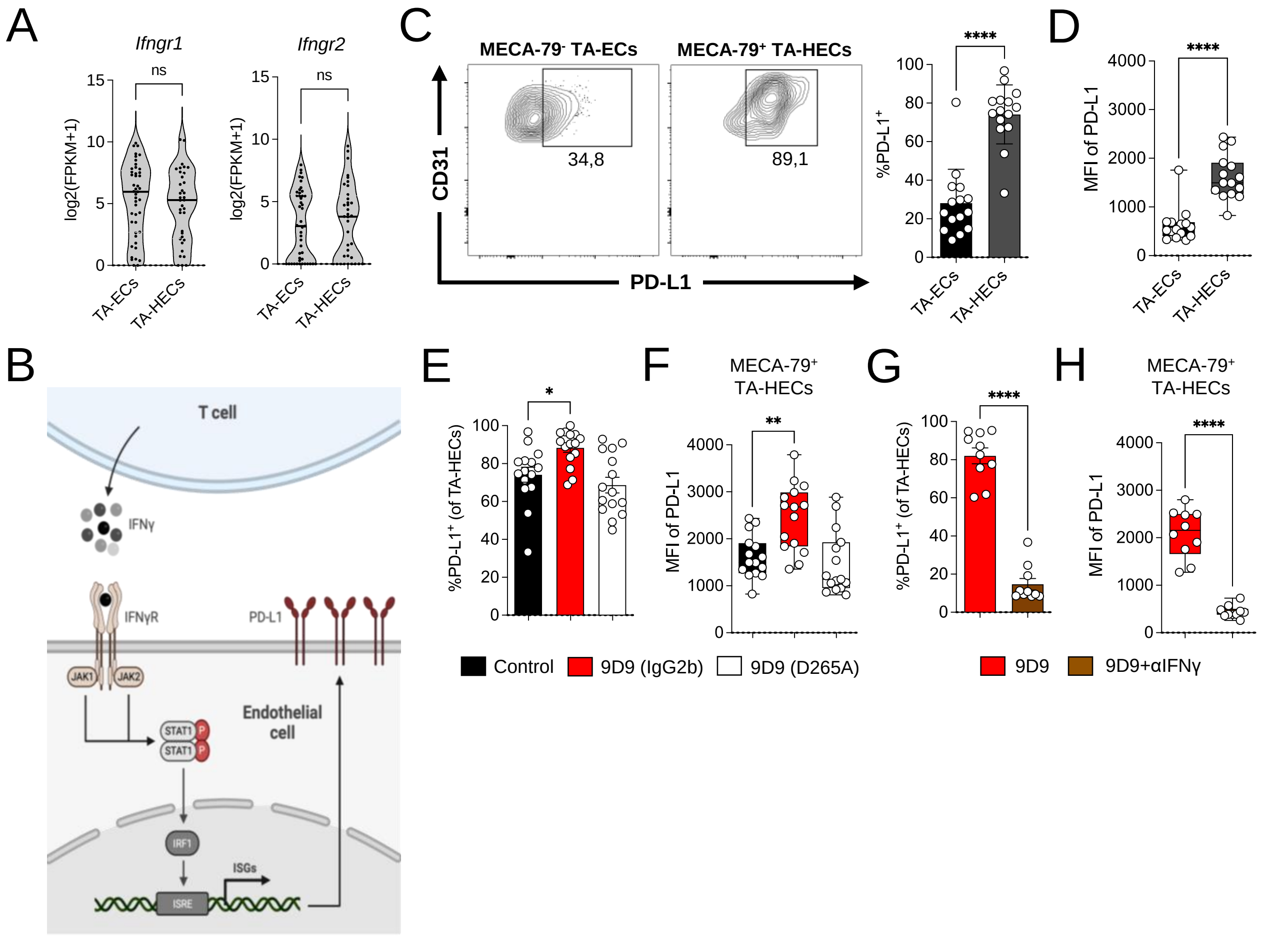

Figure S6. Anti-CTLA-4 antibodies upregulate PD-L1 on MECA-79⁺ TA-HECs via Fc- and IFNγ-dependent mechanisms, Related to Figure 3.

(A) Violin plots showing the expression distribution of selected genes in tumor-associated CD45⁻CD31^high^MECA-79⁺ HEV endothelial cells (TA-HECs) and CD45⁻CD31^high^MECA-79⁻ endothelial cells (TA-ECs). Gene expression values were obtained from single cell RNA-seq analyses of mRNA expression and calculated as fragments per kilobase of transcript per million (FKPM). y axis indicates log2 (FPKM+1) expression levels. Presented data were obtained from Asrir et al. [S4].

(B) Schematic representation of the IFNγR signaling pathway in tumor endothelial cells. IFNγR stimulation induces expression of various interferon-stimulated genes (ISGs) including *Cd274* that encodes PD-L1.

(C and D) Frequency of PD-L1⁺ cells and mean fluorescence intensity (MFI) of PD-L1 in TA-ECs and TA-HECs from untreated control MCA_{prog} tumors (day 12), quantified by flow cytometry. Representative dot plots are shown (C). Each symbol represents an individual mouse. Data were obtained from three independent experiments (total: n = 15 mice).

(E and F) Frequency of PD-L1⁺ cells and MFI of PD-L1 in TA-HECs, following indicated treatments, quantified by flow cytometry. Each symbol represents an individual mouse. Data were obtained from three independent experiments (total: 15 mice per group).

(G and H) Frequency of PD-L1⁺ cells and MFI of PD-L1 in TA-HECs, following treatment with 9D9 mouse anti-CTLA-4 antibody with or without IFNγ neutralization, quantified by flow cytometry. Each symbol represents an individual mouse. Data were obtained from two independent experiments (total: 10 mice per group).

Data are shown as mean ± SEM. All p values were determined by unpaired two-tailed Student's t test except for graphs in (E) and (F) for which p values were determined by one-way ANOVA with Tukey's multiple comparison test.

Figure S7

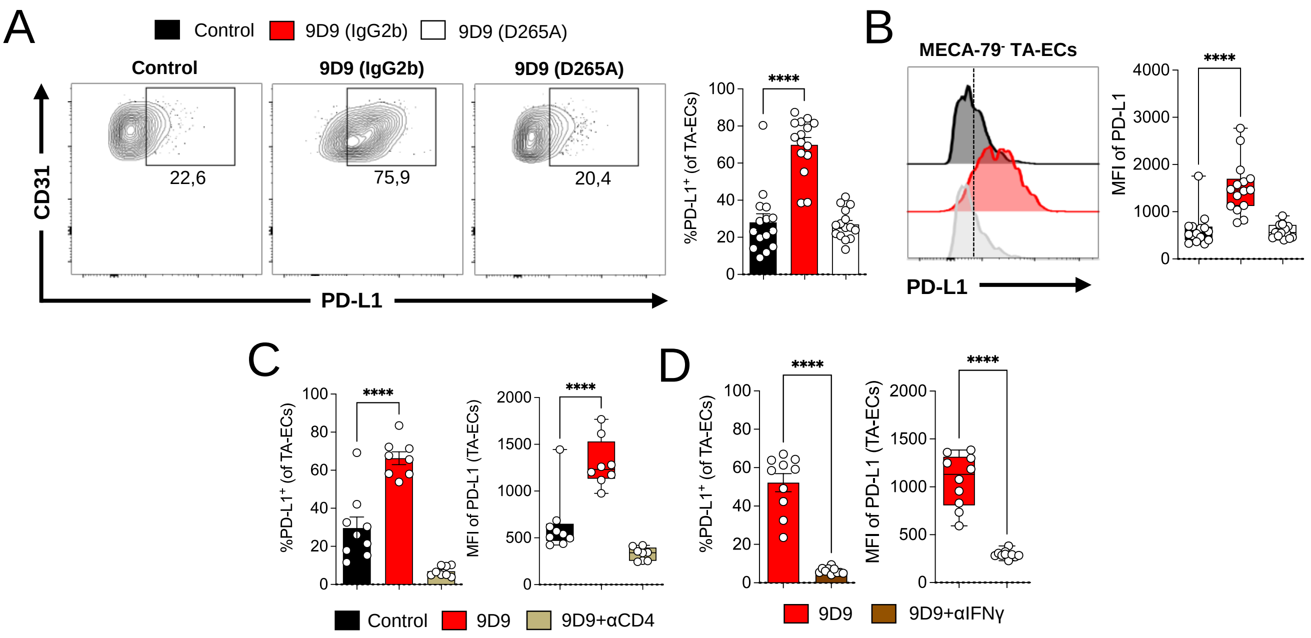

Figure S7. Anti-CTLA-4 antibodies upregulate PD-L1 on MECA-79$^-$ TA-ECs via Fc-, CD4$^+$ T cell- and IFNγ-dependent mechanisms, Related to Figure 3.

(A and B) Frequency of PD-L1$^+$ cells and mean fluorescence intensity (MFI) of PD-L1 in TA-ECs, following indicated treatments, quantified by flow cytometry. Representative dot plots and histograms are shown. Each symbol represents an individual mouse. Data were obtained from three independent experiments (total: 15 mice per group).

(C) Frequency of PD-L1$^+$ cells and MFI of PD-L1 in TA-ECs, following indicated treatments, quantified by flow cytometry. Each symbol represents an individual mouse. Data were obtained from two independent experiments (total: Control = 9 mice, 9D9 = 8 mice, 9D9 + anti-CD4 = 8 mice).

(D) Frequency of PD-L1$^+$ cells and MFI of PD-L1 in TA-ECs, following treatment with 9D9 mouse anti-CTLA-4 antibody with or without IFNγ neutralization, quantified by flow cytometry. Each symbol represents an individual mouse. Data were obtained from two independent experiments (total: 10 mice per group).

Data are shown as mean ± SEM. All p values were determined by one-way ANOVA with Tukey's multiple comparison except for graph in (D) for which p values were determined by unpaired two-tailed Student's t test.

Figure S8

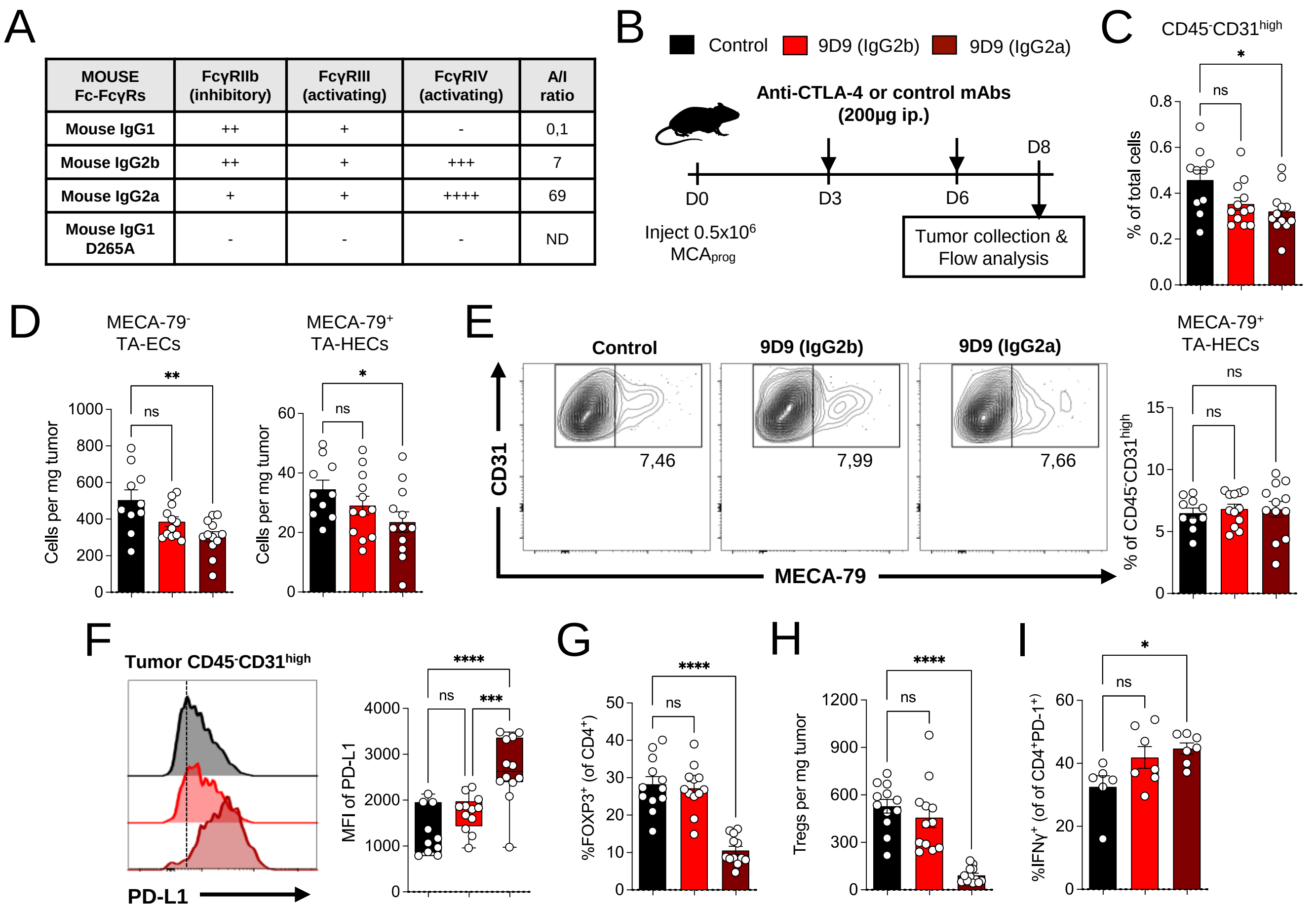

Figure S8. Fc-enhanced mouse anti-CTLA-4 antibodies have increased anti-angiogenic activity, Related to Figure 4.

(A) Table presenting the binding profiles to mouse FcγRs of the various IgGs used in the study. Relative binding affinities are proposed based on affinity constants previously assessed by surface plasmon resonance or other binding assays [S2, S3]. The relative Fc effector function of mouse IgGs is presented as the ratio of activating (FcγRIII for mouse IgG1; FcγRIV for mouse IgG2a and IgG2b) to inhibitory FcγR (FcγRIIb).

(B) Treatment schedule. ip., intraperitoneal. Isotype control antibodies were used in the control group.

(C-E) Frequency of $CD45^{-}CD31^{high}$ endothelial cells, numbers of TA-ECs and TA-HECs, and frequency of TA-HECs, following indicated treatments. Representative dot plots and histograms are shown (E). Data were obtained from three independent experiments (total: Control = 10 mice, 9D9 = 12 mice, 9D9-IgG2a = 12 mice). (F) Histograms showing expression of PD-L1 in $CD45^{-}CD31^{high}$ tumor endothelial cells following indicated treatments, quantified by flow cytometry. MFI of PD-L1 is quantified. Each symbol represents an individual mouse. Data were obtained from three independent experiments (total: Control = 10 mice, 9D9 = 12 mice, 9D9-IgG2a = 12 mice). (G and H) Frequency and number of $FOXP3^{+}$ Tregs in tumor, following indicated treatments. Each symbol represents an individual mouse. Data were obtained from three independent experiments (total: 12 mice per group). (I) Frequency of $IFN\gamma^{+}$ cells in $CD44^{+}PD\text{-}1^{+}$ $CD4^{+}$ T cells stimulated *ex vivo*, following indicated treatments. Data were obtained from two independent experiments (total: Control = 6 mice, 9D9 = 7 mice, 9D9-IgG2a = 7 mice).

Data are shown as mean ± SEM. All p values were determined by one-way ANOVA with Tukey's multiple comparison.

Figure S9

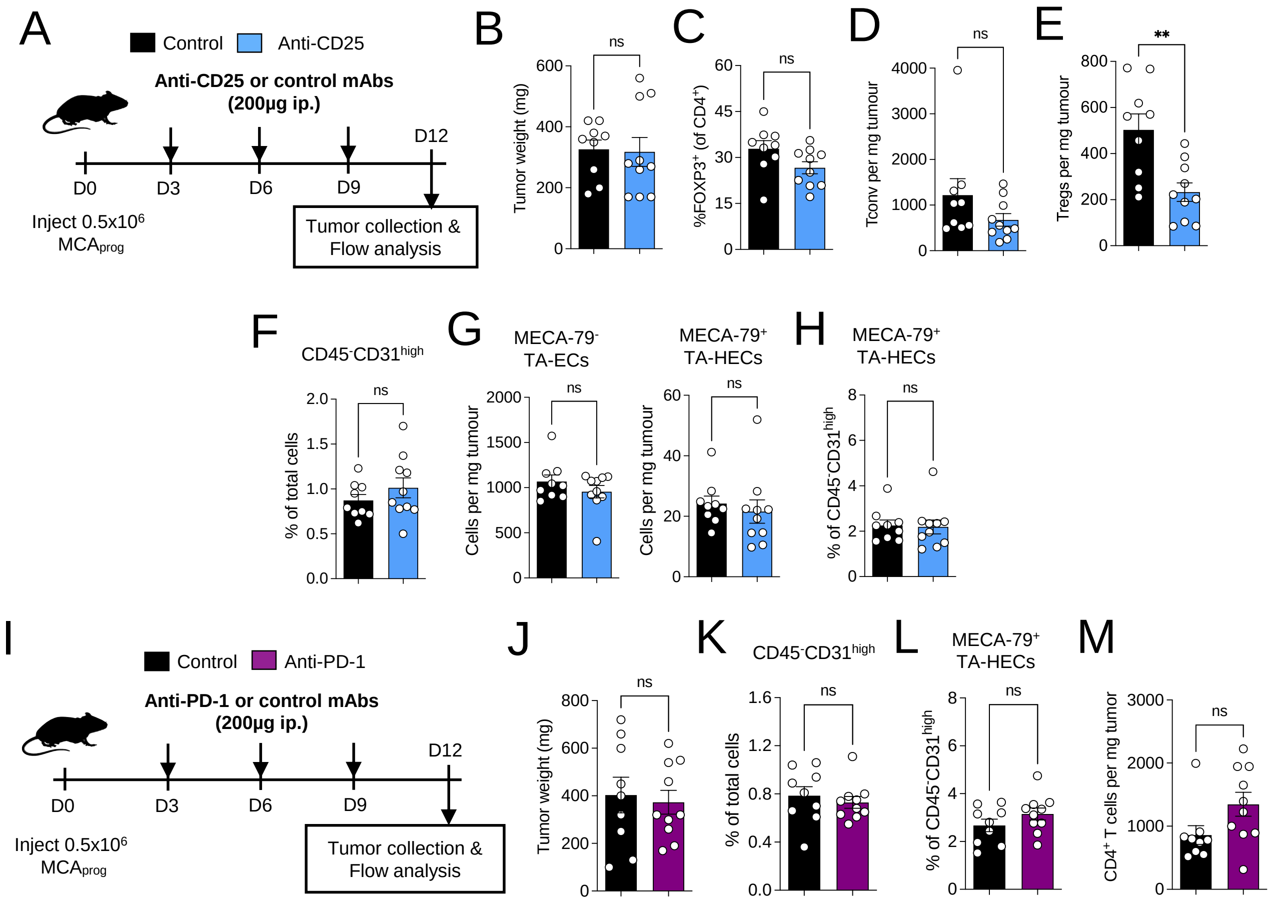

Figure S9. Anti-CD25 and anti-PD-1 antibodies do not modulate tumor endothelial cells and TA-HEVs, Related to Figure 4.

(A) Treatment schedule. ip., intraperitoneal. Isotype control antibodies were used in the control group.

(B-E) Tumor weights in individual mice, frequency of $FOXP3^+$ Tregs in tumor, and numbers of tumor-infiltrating $FOXP3^+$ Tregs and $FOXP3^-$ Tconv following indicated treatments. Each symbol represents an individual mouse. Data were obtained from two independent experiments (total: Control = 9 mice, anti-CD25 = 10 mice).

(F-H) Frequency of $CD45^-CD31^{high}$ endothelial cells, numbers of TA-ECs and TA-HECs, and frequency of TA-HECs. Each symbol represents an individual mouse. Data were obtained from two independent experiments (total: Control = 9 mice, anti-CD25 = 10 mice).

(I) Treatment schedule. ip., intraperitoneal. Isotype control antibodies were used in the control group.

(J-M) Tumor weights in individual mice, frequencies of $CD45^-CD31^{high}$ endothelial cells and TA-HECs, and number of tumor-infiltrating $CD4^+$ T cells. Data were obtained from two independent experiments (total: Control = 9 mice, anti-PD-1 = 10 mice).

Data are shown as mean ± SEM. All p values were determined by unpaired two-tailed Student's t test.

Figure S10

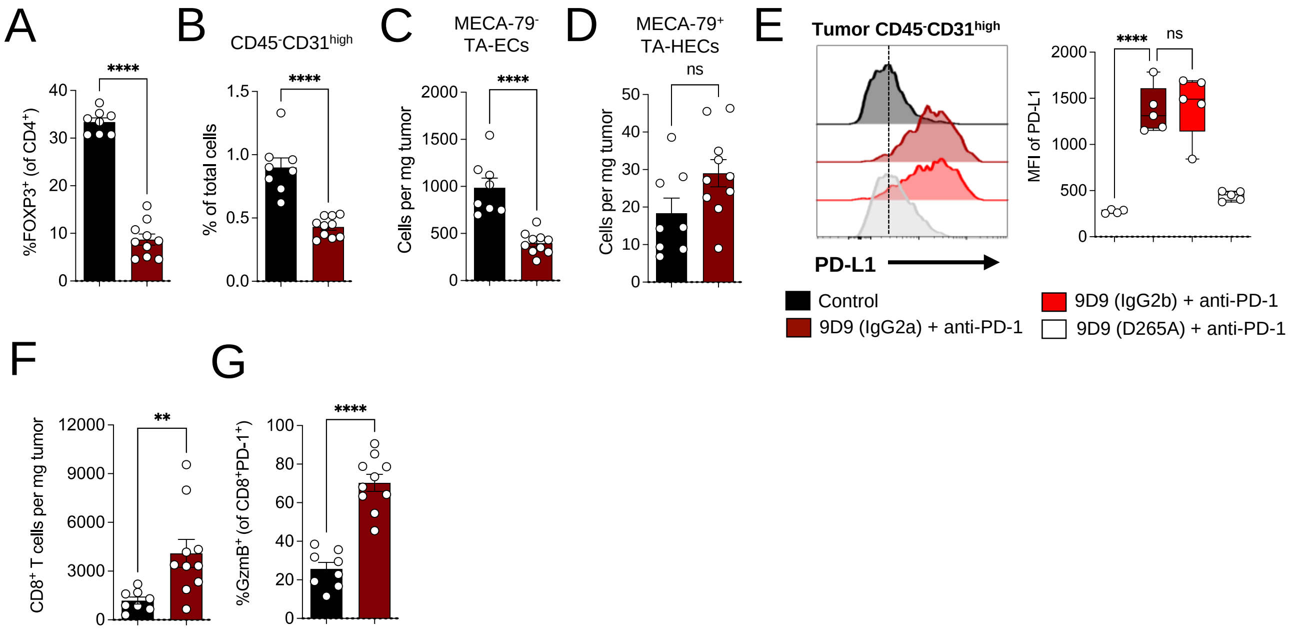

Figure S10. Concomitant PD-1 blockade prevents the pruning of TA-HEVs during treatment with Fc-enhanced mouse anti-CTLA-4 antibodies, Related to Figure 4.

(A) Frequency of FOXP3^{+} Tregs in tumor following indicated treatments. Each symbol represents an individual mouse. Data were obtained from two independent experiments (total: Control = 8 mice, 9D9-IgG2a + anti-PD-1 = 10 mice).

(B-D) Frequency of CD45^{-}CD31^{high} endothelial cells and numbers of TA-ECs and TA-HECs. Each symbol represents an individual mouse. Data were obtained from two independent experiments (total: Control = 8 mice, 9D9-IgG2a + anti-PD-1 = 10 mice).

(E) Histograms showing expression of PD-L1 in CD45^{-}CD31^{high} tumor endothelial cells following indicated treatments, quantified by flow cytometry. MFI of PD-L1 is quantified. Each symbol represents an individual mouse. Data were obtained from one experiment (total: 5 mice per group).

(F and G) Number of tumor-infiltrating CD8^{+} T cells and frequency of granzyme B (GzmB)$^{+}$ cells in tumor-infiltrating CD44^{+}PD-1^{+} CD8^{+} T cells. Each symbol represents an individual mouse. Data were obtained from two independent experiments (total: Control = 8 mice, 9D9-IgG2a + anti-PD-1 = 10 mice).

Data are shown as mean ± SEM. All p values were determined by unpaired two-tailed Student's t test except for graph in (E) for which p values were determined by one-way ANOVA with Tukey's multiple comparison test.

Figure S11

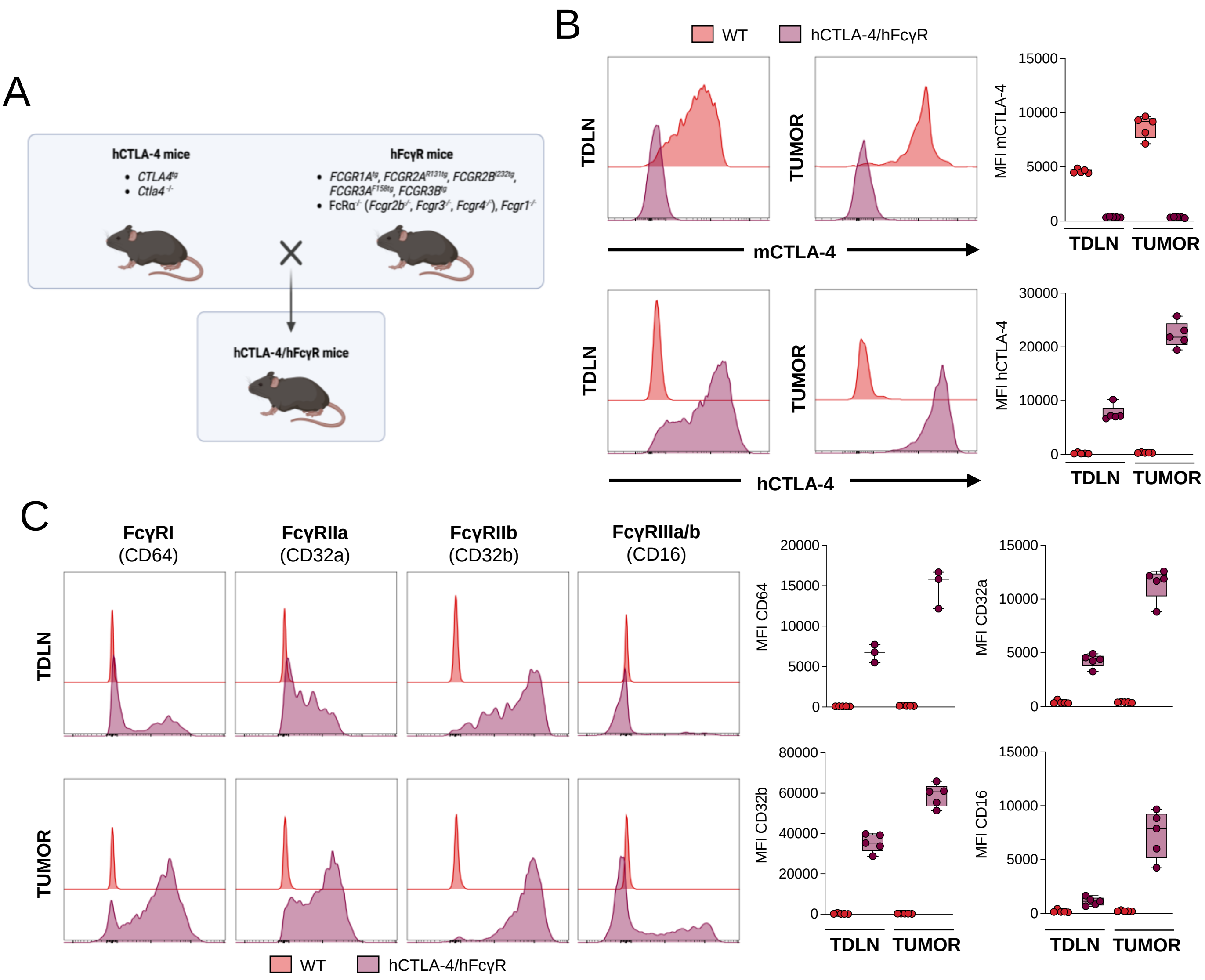

Figure S11. A humanized mouse model to study the *in vivo* activity of fully human anti-CTLA-4 antibodies, Related to Figure 5.

(A) Schematic describing the generation of humanized CTLA-4/FcγR mice. The genotypes of mouse strains used for crossing are presented.

(B) Histograms showing expression of mouse CTLA-4 (mCTLA-4) and human CTLA-4 (hCTLA-4) in FOXP3^{+} Tregs isolated from TDLN and MCA-205 tumors (day 10) of WT (red) or humanized CTLA-4/FcγR (purple) mice, quantified by flow cytometry. Mean fluorescence intensity (MFI) of mCTLA-4 and hCTLA-4 were quantified. Each symbol represents an individual mouse. Data were obtained from one experiment (n = 5 mice). (C) Histograms showing expression of human FcγRs in CD45^{+}CD11b^{+} myeloid cells isolated from TDLN and MCA-205 tumors (day 10) of WT (red) or humanized CTLA-4/FcγR (purple) mice, quantified by flow cytometry. MFI of human FcγRs were quantified. Each symbol represents an individual mouse. Data were obtained from one experiment (n = 5 mice). Data are shown as mean ± SEM.

Figure S12

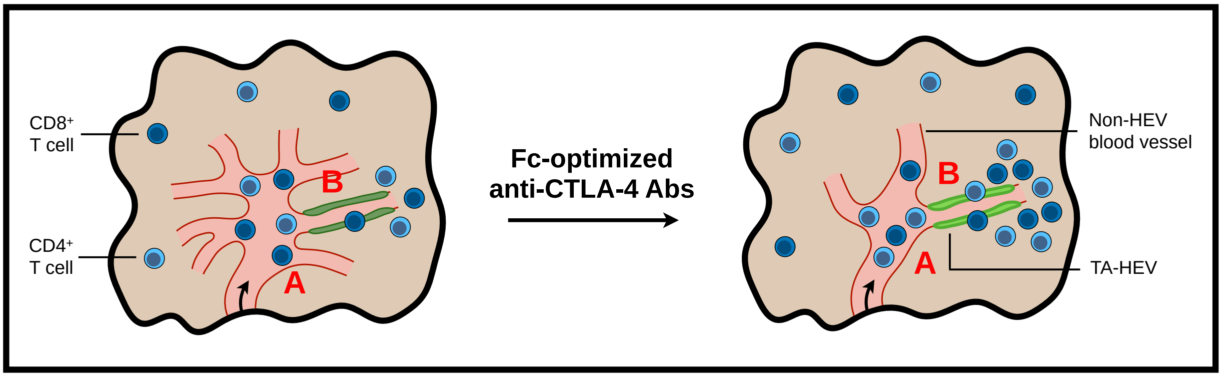

Figure S12. Model illustrating the remodeling of tumor blood vessels and the increased frequency and phenotype of TA-HEVs upon anti-CTLA-4 treatment, Related to Discussion.
(A) Modulation of the frequency of TA-HEVs upon anti-CTLA-4 treatment. The frequency of TA-HEVs among tumor blood vessels is an important parameter for TA-HEV-mediated lymphocyte entry into tumor, because it determines the frequency that a lymphocyte circulating in the tumor microvasculature encounters a MECA-79$^+$ TA-HEC with high recruitment capacity vs a MECA-79$^-$ TA-EC with poor recruitment capacity. In the MCA_{prog} tumor model, treatment with anti-CTLA-4 mAbs 9D9 and 9H10 increased the frequency of MECA-79$^+$ TA-HECs by reducing the numbers of MECA-79$^-$ TA-ECs (~ 50% reduction) while maintaining a constant number of TA-HECs. Remodeling of tumor blood vessels and anti-angiogenic effects of anti-CTLA-4 mAb 9D9 were not observed in the presence of antibodies neutralizing IFNγ, a potent anti-angiogenic cytokine produced by Th1-like CD4$^+$ T cells. Pruning of dysfunctional non-HEV tumor blood vessels upon anti-CTLA-4 treatment will lead to an increased frequency of TA-HEVs. Thus, more lymphocytes will circulate in TA-HEVs and interact with MECA-79$^+$ TA-HECs. This will result in increased numbers of lymphocytes entering tumors through TA-HEVs. (B) Modulation of the phenotype of TA-HEVs upon anti-CTLA-4 treatment. TA-HEVs exhibit a bi-functional phenotype (shown in dark green in the image on the left) characterized by the co-expression of MECA-79$^+$ antigens (counter-receptors for lymphocyte CD62L) and endothelial CD62P (receptor for lymphocyte PSGL-1), that are critical for lymphocyte rolling in TA-HEVs and increased infiltration of CD4$^+$ and CD8$^+$ T cells into tumors during cancer immunotherapy [S4]. Anti-CTLA-4 mAb 9D9 increases the expression levels (MFI) of MECA-79$^+$ antigens and CD62P on MECA-79$^+$ TA-HECs in an Fc-dependent manner in the MCA_{prog} tumor model. Increased levels of MECA-79 and CD62P on TA-HEVs (shown in light green in the image on the right) are likely to result in an increased functionality of TA-HEVs upon anti-CTLA-4 treatment. Another critical parameter influencing lymphocyte recruitment through TA-HEVs, is the capacity of TA-HEVs to mediate lymphocyte arrest and extravasation, through the presentation at their surface of tissue-derived chemokines, such as the IFNγ-regulated chemokines CXCL9, CXCL10 and CCL21 [S5-S7]. Treatment with anti-CTLA-4 mAbs increased the proportion of IFNγ-producing CD4$^+$ T cells and resulted in upregulation of the IFNγ-inducible PD-L1 protein. Thus, upregulation of IFNγ-regulated chemokines is also likely to occur rapidly upon treatment and could contribute to an increased functionality of TA-HEVs by increasing chemokine-dependent lymphocyte arrest and transmigration. This later mechanism could be particularly relevant for the Fc-enhanced anti-CTLA-4 mAb 9D9 IgG2a which is a potent inducer of IFNγ-producing CD4$^+$ T cells.

Supplemental References

[S1] Simpson, T.R., Li, F., Montalvo-Ortiz, W., Sepulveda, M.A., Bergerhoff, K., Arce, F., Roddie, C., Henry, J.Y., Yagita, H., Wolchok, J.D., et al. (2013). Fc-dependent depletion of tumor-infiltrating regulatory T cells co-defines the efficacy of anti-CTLA-4 therapy against melanoma. J Exp Med *210*, 1695–1710. https://doi.org/10.1084/JEM.20130579.

[S2] Nimmerjahn, F., and Ravetch, J. V. (2005). Divergent immunoglobulin g subclass activity through selective Fc receptor binding. Science *310*, 1510–1512. https://doi.org/10.1126/SCIENCE.1118948.

[S3] Nimmerjahn, F., Bruhns, P., Horiuchi, K., and Ravetch, J. V. (2005). FcgammaRIV: a novel FcR with distinct IgG subclass specificity. Immunity *23*, 41–51. https://doi.org/10.1016/J.IMMUNI.2005.05.010.

[S4] Asrir, A., Tardiveau, C., Coudert, J., Laffont, R., Blanchard, L., Bellard, E., Veerman, K., Bettini, S., Lafouresse, F., Vina, E., et al. (2022). Tumor-associated high endothelial venules mediate lymphocyte entry into tumors and predict response to PD-1 plus CTLA-4 combination immunotherapy. Cancer Cell *40*, 318-334.e9. https://doi.org/10.1016/J.CCELL.2022.01.002.

[S5] Peske, J.D., Thompson, E.D., Gemta, L., Baylis, R.A., Fu, Y.X., and Engelhard, V.H. (2015). Effector lymphocyte-induced lymph node-like vasculature enables naive T-cell entry into tumours and enhanced anti-tumour immunity. Nat Commun *6*. https://doi.org/10.1038/ncomms8114.

[S6] Spranger, S., Dai, D., Horton, B., and Gajewski, T.F. (2017). Tumor-Residing Batf3 Dendritic Cells Are Required for Effector T Cell Trafficking and Adoptive T Cell Therapy. Cancer Cell *31*, 711-723.e4. https://doi.org/10.1016/J.CCELL.2017.04.003.

[S7] Dangaj, D., Bruand, M., Grimm, A.J., Ronet, C., Barras, D., Duttagupta, P.A., Lanitis, E., Duraiswamy, J., Tanyi, J.L., Benencia, F., et al. (2019). Cooperation between Constitutive and Inducible Chemokines Enables T Cell Engraftment and Immune Attack in Solid Tumors. Cancer Cell *35*, 885-900.e10. https://doi.org/10.1016/J.CCELL.2019.05.004.
